# Unveiling Varied Cell Death Patterns in Lung Adenocarcinoma Prognosis and Immunotherapy Based on Single‐Cell Analysis and Machine Learning

**DOI:** 10.1111/jcmm.70218

**Published:** 2024-11-27

**Authors:** Zipei Song, Weiran Zhang, Miaolin Zhu, Yuheng Wang, Dingye Zhou, Xincen Cao, Xin Geng, Shengzhe Zhou, Zhihua Li, Ke Wei, Liang Chen

**Affiliations:** ^1^ Department of Thoracic Surgery The First Affiliated Hospital of Nanjing Medical University Nanjing China; ^2^ Department of Oncology The Affiliated Cancer Hospital of Nanjing Medical University and Jiangsu Cancer Hospital and Jiangsu Institute of Cancer Research Nanjing China

**Keywords:** immunotherapy response, lung adenocarcinoma (LUAD), machine learning, prognosis, programmed cell death, single‐cell RNA‐seq

## Abstract

Programmed cell death (PCD) pathways hold significant influence in the etiology and progression of a variety of cancer forms, particularly offering promising prognostic markers and clues to drug sensitivity for lung adenocarcinoma (LUAD) patients. We employed single‐cell analysis to delve into the functional role of PCD within the tumour microenvironment (TME) of LUAD. Employing a machine learning framework, a PCD‐related signature (PCDS) was constructed utilising a comprehensive data set. The PCDS exhibited superior prognostic performance compared with the 140 previously established prognostic models for LUAD. Subsequently, patients were stratified into high‐risk and low‐risk groups based on their risk scores derived from the PCDS, with the high‐risk group exhibiting significantly lower overall survival (OS) rates than the low‐risk group. Furthermore, the risk subgroups were compared for differences in pathway enrichment, genomic alterations, tumour immune microenvironment (TIME), immunotherapy and drug sensitivity. The low‐risk group displayed a more inflamed TIME, potentially leading to a more favourable response to immunotherapy. For the high‐risk group, potential effective small molecule drugs were identified, and the drug sensitivity were evaluated. Immunohistochemistry and quantitative real‐time polymerase chain reaction assays (qRT‐PCR) confirmed notable upregulation of the expression levels of PCD‐associated genes MKI67, TYMS and LYPD3 in LUAD tissues. In vitro experimental findings demonstrated a marked decrease in the proliferative and migratory capacities of LUAD cells upon knockdown of MKI67. Conclusively, we successfully constructed the PCDS, providing important assistance for prognosis prediction and treatment optimisation of LUAD patients.

## 1 Introduction

Lung cancer represents a prevalent malignancy worldwide and remains a prominent contributor to cancer‐related mortality [1]. Lung adenocarcinoma (LUAD) comprises roughly 40 percentage of all lung cancer diagnoses [2]. For a significant portion of patients diagnosed with early‐stage LUAD, surgical excision stands as the most effective treatment modality. Conversely, certain advanced‐stage LUAD patients may find benefit in chemotherapy augmented with targeted therapies [3, 4, 5]. Despite strides made in treatment modalities, the identification of innovative prognostic indicators and therapeutic agents for LUAD is paramount.

Programmed cell death (PCD) is a cell death process that occurs through precise regulation and active involvement of the cellular activities [6]. PCD is responsible for removing cells that are not functionally necessary, infected or potentially cancerous. Its importance lies in homeostasis, defending against pathogens, managing cancer and other pathological conditions [7, 8, 9]. PCD has long been acknowledged as a crucial mechanism in controlling tumour development. Tumour biology can be influenced by PCD, as it can either support or hinder tumour progression [10]. The capacity of tumour cells to evade or withstand PCD is closely related to their uncontrolled growth and malignancy. Recent findings reveal that PCD exerts a crucial influence in shaping the immunosuppressive tumour microenvironment (TME) and ultimately determining the success or failure of cancer treatment outcomes. PCD also contributes to the modulation of effector cells and regulatory immune cells, actively participating in contributing to the antitumour immune response within the TME [11].

PCD encompasses multiple cell death mechanisms, and here, we list 13 main mechanisms, including apoptosis, autophagy, pyroptosis, necroptosis, netotic cell death, entotic cell death, lysosome‐dependent cell death, parthanatos, oxeiptosis, ferroptosis, cuproptosis, alkaliptosis and disulfidptosis [9, 10, 12, 13]. Apoptosis stands as one of the earliest and universally acknowledged manifestations of PCD. The TME has acknowledged apoptosis resulting from therapeutic interventions or cytotoxic immune cells as the primary mechanism for tumour cell eradication [14, 15]. Autophagy, on the other hand, represents a survival strategy utilised by eukaryotic cells in response to nutrient scarcity. This process facilitates the recycling of cellular nutrients and the preservation of organelles, thereby mitigating premature cell demise due to nutrient deprivation [16, 17, 18]. However, pyroptosis, a lytic and inflammatory form of PCD, is typically initiated by the activation of inflammasomes and executed by gasdermin protein. As an immunogenic cell death pathway, pyroptosis presents a novel therapeutic cancer treatment approach eradication by simultaneously triggering cellular demise and igniting robust antitumour immune responses [19]. Conversely, ferroptosis stems from an excessive accumulation of intracellular reactive oxygen species (ROS), leading to iron‐mediated peroxidation of polyunsaturated fatty acids within the cell membrane, ultimately causing membrane disruption [20, 21]. Exploiting ferroptosis in cancer cells within the TME has emerged as an alternative therapeutic avenue for cancer treatment [22, 23, 24]. A key characteristic of cuproptosis is the abnormal accumulation of intracellular copper ions, which is closely linked to mitochondrial respiration and the fatty acid metabolism pathway. It may be involved in the progression of various types of cancer [25]. Disulfidptosis represents a novel modality of PCD that typically occurs under glucose‐starved conditions. Its triggering factor is the excessive accumulation of cysteine, leading to disulfide stress [12, 13, 26]. In addition, other forms of PCD have been extensively discussed in LUAD [27]. However, the comprehensive interplay and interconnection between these 13 distinct forms of PCD and the anticancer immune response in LUAD remains unclear. While there has been an improved understanding of PCD in lung cancer, further elucidation is needed regarding how PCD influences the occurrence and progression of lung cancer. In particular, the correlation between PCD and the prognosis and treatment response of lung cancer patients has not been fully revealed.

Single‐cell RNA sequencing (ScRNA‐seq), a high‐efficiency sequencing technology, can be used to studied the individual cell level gene expression. Traditional bulk RNA sequencing combines a large number of cells for analysis, which masks the heterogeneity among different cells. In contrast, scRNA‐seq helps to uncover cellular heterogeneity, discover novel cell types and subtypes, investigate cell developmental trajectories, explore cell–cell interactions and signalling pathways, as well as study disease mechanisms [28, 29, 30]. Biomarkers play a crucial role in guiding treatment strategies, improving treatment outcomes, and contributing to enhanced patient survival rates and quality of life in the field of academic medicine [31, 32, 33, 34]. Despite the identification of various cancer biomarkers through bioinformatics approaches, the reliability and clinical translational potential of these biomarkers still require further investigation due to the lack of comparative analyses and clinical validations among different biomarkers [35, 36, 37]. A PCD‐related signature (PCDS) was developed in LUAD through the analysis of scRNA‐seq data and bulk RNA sequencing data using 101 machine learning (ML) algorithms in this study. Subsequently, the association of the PCDS score with the prognosis, tumour immune microenvironment and treatment response was investigated in LUAD patients. The findings of our research introduce a novel prognostic biomarker for LUAD, thereby facilitating treatment decision‐making. Additionally, it provides insights into potential mechanisms of PCD in LUAD.

## 2 Materials and Methods

### 2.1 Data Acquisition and Preprocessing

The scRNA‐seq data set pertaining to LUAD, encompassing a total of 12 distinct LUAD samples, was retrieved from the GEO repository, (URL: https://www.ncbi.nlm.nih.gov/geo/; Accession Number: GSE150938). The signature was established by acquiring transcriptome data and clinical data for 1393 LUAD patients from public databases. Samples without clinical information and those with an overall survival (OS) of 0 were omitted from the subsequent analysis phase. The training cohort consisted of 512 LUAD patients from The Cancer Genome Atlas (TCGA) database (URL: https://portal.gdc.cancer.gov/), from which bulk RNA‐seq data, mutation data and clinical characteristics were obtained. To validate the findings, transcriptional signatures and matched clinical outcomes of 881 LUAD patients were downloaded from four GEO data sets: GSE30219 (*n* =  83), GSE31210 (*n* = 226), GSE42127 (*n* =  131) and GSE68465 (*n* =  441). Furthermore, RNA‐seq data of 288 normal lung tissues were downloaded from the Genotype‐Tissue Expression (GTEx) database (URL: https://gtexportal.org/home/) for comparative analysis. For the evaluation of immunotherapy response, transcriptome data from the GSE126044 immunotherapy data set were obtained, comprising 16 NSCLC patients who received anti‐PD‐1 immunotherapy (including 5 responders and 11 nonresponders). In order to maintain consistency and comparability across data sets, all expression data were standardised to a common scale of transcripts per million (TPM), with the exception of differential analysis that specifically required counts data format. To account for any confounding effects, the ‘combat’ function embedded within the ‘sva’ R package was employed to remove potential batch effects. To ensure a more appropriate scale for statistical analysis, a logarithmic transformation using base 2 was applied to all data points prior to the initiation of the analysis.

For assembling the PCD‐related gene list, we collected information on 13 distinct PCD patterns from esteemed scientific references, encompassing GSEA gene sets, KEGG, review papers and handpicked data [34]. Once duplicate genes were removed, a cumulative count of 2088 genes linked to PCD was incorporated for further examination. In the case of 2088 genes associated with PCD, a Univariate Cox regression analysis was conducted utilising the transcriptome and clinical information from the TCGA‐LUAD data set. This analysis resulted in the identification of 455 PCD‐related genes that exhibited significant prognostic value, with statistical significance set at *p* < 0.05.

### 2.2 The Single‐Cell Analysis Process

In the analysis of the GSE150938 data set, the scRNA‐seq data from 12 LUAD samples were processed using the Seurat package (version 5.0.1) of the R software (version 4.3.0). Cell quality control was initially conducted to filter out low‐quality genes and cells based on several criteria including the expression of genes in at least 3 cells, a range of 300–7000 genes expressed per cell, mitochondrial gene expression below 10%, and red blood cell‐related gene expression below 3%, as well as cells with fewer than 100,000 UMIs. Following these quality control steps, 45,761 high‐quality cells were selected. Subsequently, the data underwent normalisation, scaling, and identification of highly variable genes using the ‘NormalizeData’, ‘ScaleData’ and ‘FindVariableFeatures’ functions, respectively. To mitigate potential batch effects, we employed the ‘RunHarmony’ function. After this, we conducted a Principal Component Analysis (PCA) to identify suitable anchor points, which were then subjected to t‐distributed Stochastic Neighbour Embedding (t‐SNE) for further dimensionality reduction. For cluster analysis, we utilised the ‘FindNeighbors’ and ‘FindClusters’ functions, setting the resolution parameter to 0.5, resulting in the identification of 16 cell clusters. The identification of marker genes for each cluster was achieved through the utilisation of the ‘FindAllMarkers’ function, which compared cells from a particular cluster with cells from all other clusters. This enabled the identification of differentially expressed genes (DEGs) for each cluster, based on established thresholds, where the adjusted *p* value was less than 0.05 and the log2 > 0.25. Cell types were annotated and identified according to the distinctive marker genes of each cluster. For the analysis of PCD activity, PCD activity scores were assigned to individual cells by employing the ‘AUCell’ and ‘AddModuleScore’ R package. Subsequently, cells were categorised into groups with high and low PCD‐AUC based on their median AUC scores. The ‘ggplot2’ R package was utilised to visualise these groups. To quantitatively evaluate the differences in cellular communication patterns between the groups characterised by either high or low PCD‐AUC scores, the ‘cellchat’ R software was employed [38, 39].

### 2.3 The Selection of Key Genes

Utilising the ‘findMarker’ tool, we pinpointed genes displaying significant variations between elevated and diminished levels PCD‐AUC groups (threshold set at |log2FC| > 0.7 and *p* < 0.05). Subsequently, we conducted a Spearman correlation analysis to select the top 150 genes exhibiting the most intense correlation with the PCD. Subsequently, the genes with strong correlations were combined with those showing notable differential expression, culminating in 374 genes in total, laying the groundwork for creating future predictive models. In the GTEx database, RNA‐seq data were downloaded from 288 normal lung tissues to compensate for the limited quantity of these samples in the TCGA data set. Subsequently, these data were integrated with the TCGA data set to create a final data set consisting of 346 normal lung tissue samples and 513 LUAD samples. These data were normalised using a log2(count+1) transformation and batch effects were removed. Transcriptomic data for 374 PCD‐related genes were obtained from this data set, among which a total of 145 PCD‐related DEGs were identified between LUAD and normal tissues (*p* < 0.05, |log2FC| > 1.5). Following this, a univariate Cox regression analysis was conducted utilising transcriptomic and clinical information from the TCGA‐LUAD data set, leading to the identification of 38 PCD‐related genes with substantial predictive importance (*p* < 0.05). Owing to the loss of genes in the batch elimination phase of later TCGA‐LUAD and GEO data, the expression levels of 25 genes were ultimately employed in our model construction.

### 2.4 Construction of PCDS Through 101 Combinations of Machine Learning Algorithms

To create a robust and dependable prognostic signature named as PCDS, a combination of 10 ML algorithms and 101 algorithm combinations were integrated, including Lasso, Enet, stepwise Cox, plsRcox, SuperPC, RSF, CoxBoost, GBM, Ridge and survival‐SVM. The signature generation process is outlined as follows: (a) A machine learning computational framework was employed, utilising 101 algorithm combinations, to construct a total of 101 prognostic models based on 25 key genes. (b) These models were assessed using four validation data sets (GSE30219, GSE31210, GSE42127 and GSE68465). (c) The C‐index for each model was calculated applying above four validation data sets, to select the optimal model with the highest mean value of C‐index. (d) Utilising the chosen model, the genes participating in the were identified, followed by the computation of risk scores for each sample. Subsequently, in every data set, patients were classified into high‐ and low‐risk categories based on their median risk scores. The stringent procedure guaranteed the creation of a dependable and strong predictive marker, which was validated across multiple data sets.

### 2.5 Assessment of PCDS and Construction of a Predictive Nomogram

The ‘survminer’ R package was employed to conduct K–M curve analysis, aiming to investigate potential differences in OS and progression‐free survival (PFS) across the two groups, each marked by varying levels of risk sores. To assess the predictive accuracy of PCDS, alongside age, gender and disease stage in predicting the OS in LUAD patients, receiver operating characteristic (ROC) analyses were performed using the ‘timeROC’ package, concurrently with the area under the curve (AUC) for these predictive factors were computed and analysed. For dimensionality reduction and visualisation of sample distribution disparities between the two groups categorised by different risk sore levels, PCA was employed. Moreover, the correlations between PCDS and various clinical characteristics, including age, gender and stage, were explored. The ‘CompareC’ package was used to calculate and compare the C‐index values of risk scores and clinical features in the TCGA, GSE30219, GSE31210, GSE42127 and GSE68465 data sets. Subsequently, a total of 140 published signatures for prognostic prediction of LUAD patients were obtained from a literature search on PubMed. And then the C‐index of PCDS and these signatures were compared in the TCGA, GSE30219, GSE31210, GSE42127 and GSE68465 data sets. Ultimately, a nomogram was developed by merging risk assessments with clinical factors, targeting the prediction of LUAD patients' overall survival at 1, 3 and 5 years. The nomogram we developed incorporates the TNM staging system. Given that the GSE31210 and GSE42127 data sets did not contain clinical information related to TNM staging, and GSE68465 lacked clinical staging information, we opted to utilise the TCGA‐LUAD data set as the training set and the GSE30219 data set as the testing set to validate the nomogram. For evaluating the predictive precision and confirming the efficacy of this nomogram, the calibration curve is used, together with decision curve analysis (DCA) and ROC curves, were graphically represented.

### 2.6 Functional Enrichment Analysis

Investigating the fundamental biological processes linked to PCDS, GSVA and GSEA were performed based on the MsigDB database, by the use of R packages including ‘limma’, ‘GSVA’, ‘ClusterProfiler’, ‘org.Hs.eg.db’ and ‘GseaVis’. Enrichment analysis of the DEGs between the two groups with different risk score levels was conducted applying the Metascape website (https://metascape.org/), and the results were presented through bar plots and pathway‐based network diagrams [40]. Enrichment scores of each sample were calculated according to enriched pathways, and then t‐SNE dimensionality reduction was applied for results visualisation.

### 2.7 Genomic Variant Analysis

A study utilising GISTIC 2.0 (https://gatk.broadinstitute.org) was performed to pinpoint areas of the genome that are often amplified or removed. Furthermore, the tumour mutation burden (TMB) was assessed using the ‘maftools’ R package [41]. Patients with LUAD were categorised into high and low TMB groups according to their median TMB scores. Finally, a comparison was made between the prognoses of these two groups by integrating the clinical information from TCGA.

### 2.8 Assessing the Relationship Between PCDS and Immune Infiltration Characteristics

The ‘ESTIMATE’ R package was utilised to calculate the immune score, stromal score and ESTIMATE score for every patient. The aim is to evaluate the possible variances in the TIME between groups categorised as high‐risk and low‐risk, a correlation analysis was conducted to assess the connection between these scores, the purity of the tumour and the risk score. A total of eight unique algorithms, namely TIMER, xCELL, CIBERSORT, CIBERSORT‐ABS, quanTIseq, MCP‐counter, EPIC and ssGSEA, were implemented to compare the differences in TIME between the high‐risk and low‐risk groups [42, 43]. Subsequently, the correlation between PCDS scores and the cancer immune cycle was explored. Finally, comparative analysis of immune checkpoint (IC) expression and immune modulators was performed to explore the possible effectiveness of PCDS in forecasting reactions to immunotherapy.

### 2.9 Immunotherapeutic Response Prediction

Investigating how PCDS might predict outcomes in immunotherapy, we accessed and used the Tumor Immune Dysfunction and Exclusion (TIDE) website (https://tide.dfci.harvard.edu/) on the internet. Reduced TIDE scores frequently correlate with increased Positive reactions to therapy involving immune checkpoint inhibitors (ICIs). Genetic transcript information from the GSE126044 immunotherapy collection, which includes 16 patients with non‐small‐cell lung cancer (NSCLC) (comprising 5 responders and 11 nonresponders) who underwent anti‐PD‐1 immune therapy, were obtained. Following this, the data set facilitated the computation of PCDS risk scores to forecast immunotherapy outcomes. Ultimately, the immune phenotype scores (IPS) for LUAD patients were sourced from The Cancer Immunome Atlas (TCIA) database (https://tcia.at/home) to forecast their possible reaction to immune checkpoint blockade (ICB) drugs.

### 2.10 Predicting the Efficacy of Potential Small‐Molecule Drugs and Drug Sensitivity

For the evaluation of drug sensitivity and further identification of potentially effective small‐molecule drugs for LUAD patients with different risk score levels, a ridge regression model was generated based on the TCGA transcriptome data. The data used for this analysis including drug sensitivity AUC data from the Cancer Therapeutics Response Portal database (CTRP 2.0, https://portals.broadinstitute.org/ctrp.v2.1/) and the PRISM database (https://www.theprismlab.org/), as well as the cell line expression profiles from the Cancer Cell Line Encyclopedia database (CCLE, https://sites.broadinstitute.org/ccle/). By assessing the Spearman correlation between risk scores and AUC scores (CTRP 2.0: Spearman's *r* < −0.4; PRISM: Spearman's *r* < −0.35), a total of 11 CTRP 2.0‐derived compounds and 24 PRISM‐derived compounds were obtained. To explore the interactions of SB‐743921 and Ispinesib with MKI67 and TYMS proteins, respectively. The two‐dimensional structures of small‐molecule ligands were retrieved from the PubChem database, accessible at https://pubchem.ncbi.nlm.nih.gov/. Subsequently, their three‐dimensional structures were constructed utilising the Chem Office software. Then, the RCSB PDB database (https://www.rcsb.org/) was utilised to screen for crystal structures with high resolution (protein targets) that serve as molecular docking receptors. The protein structures were further processed using the PyMOL software, including operations such as dehydration and dephosphorylation. The compounds were subjected to energy minimisation employing the Molecular Operating Environment (MOE) 2019 software, while the target proteins underwent preprocessing to pinpoint active pockets. Subsequently, a molecular docking was carried out utilising MOE 2019. Based on the computed binding energies, the outcomes of these interactions were vividly illustrated using PyMOL and Discovery Studio software. To gain insights into the half‐maximal inhibitory concentration (IC50) values of widely utilised anticancer drugs, we harnessed the Cancer Drug Sensitivity Genomics of Cancer Cell Lines (GDSC) database (accessible at https://www.ancerrxgene.org/) Leveraging the R package ‘pRRophetic’, we evaluated drug sensitivity in both high‐ and low‐risk groups. For predictive outcome estimation, we employed ridge regression analysis, coupled with 10‐fold cross‐validation, to assess the predictive accuracy comprehensively.

### 2.11 Immunohistochemistry

The collection of tissue samples obtained ethics approval from the Medical Ethics Committee of the First Affiliated Hospital of Nanjing Medical University. These samples, including LUAD samples and paratumour samples, were collected on the day of the surgery for subsequent immunohistochemical staining. Immunohistochemistry (IHC) were performed on PFFE tissue sections by incubating with following primary antibodies against MKI67 (Proteintech, 1:200), TYMS (Proteintech, 1:200) and LYPD3 (Proteintech, 1:200) overnight at 4°C. Subsequently, secondary antibodies conjugated with horseradish peroxidase (HRP) (maxim) were applied and incubated for a duration of 30 min at a temperature of 37°C. The sections were then stained with DAB (3,3′‐diaminobenzidine) and counterstained with haematoxylin for visualisation.

### 2.12 Cell Lines Culture

The 16HBE human bronchial epithelial cell line, as well as two distinct human lung adenocarcinoma cell lines (A549 and H1299), were procured from the esteemed Institute of Biochemistry and Cell Biology, belonging to the Chinese Academy of Sciences. These cells were cultivated in a specific medium, namely DMEM (Gibco; Thermo Fisher Scientific Inc.), which was enriched with 10% fetal bovine serum (FBS) (also sourced from Gibco; Thermo Fisher Scientific Inc.) and supplemented with 1% penicillin–streptomycin. To maintain optimal growth conditions, the culture environment was rigorously controlled, with a constant temperature of 37°C and a regulated CO_2_ concentration.

### 2.13 Cell Transfection

KeyGEN (Nanjing, China) was responsible for the synthesis of short interfering RNAs (siRNAs). The transfection processes utilised Lipofectamine 2000 (Invitrogen, USA), adhering to the guidelines provided by the manufacturer. Evaluating the efficiency of transfection performed 48 h post transfection, employing quantitative real‐time PCR (qRT‐PCR). The results targeted are listed below:

**Table:** 

si‐MKI67‐1#	Sequence (5′ → 3′)
Sense	AGCCGAAGUCAACAUGAUAUU
Antisense	UAUCAUGUUGACUUCGGCUGA

si‐MKI67‐2#	Sequence (5′ → 3′)
Sense	CGAAGGUUCUCAUGCAGAAUC
Antisense	UUCUGCAUGAGAACCUUCGCA

### 2.14 Isolation of RNA and Quantitative Real‐Time Polymerase Chain Reaction Assays (qRT‐PCR)

Following the guidelines provided by the manufacturer, we extracted total RNA from cells utilising the TRIzol reagent (Invitrogen; Thermo Fisher Scientific Inc.). Following this, the process of reverse transcription was executed utilising the PrimeScript Reverse Transcription Kit (Ta kara Bio, Incorporated.).

For qRT‐PCR, the Archimed‐X4 Real‐Time PCR System (Applied ARCHIMED X; RocGene Technology Inc.) was employed. The qRT‐PCR protocol comprised an initial denaturation step at 95°C for 30 s, followed by 40 iterative cycles. Each cycle involved a brief denaturation at 95°C for 10 s, immediately followed by an annealing phase at 60°C for 30 s. To quantify the relative mRNA expression levels, the 2^−ΔΔCt^ method was employed, with GAPDH serving as the internal control for normalisation purposes. The entire qRT‐PCR process was replicated three times to ensure reproducibility. The specific gene primers utilised in this study are listed:

**Table:** 

GAPDH	Sequence (5′ → 3′)
Forward Primer	TGTGGGCATCAATGGATTTGG
Reverse Primer	ACACCATGTATTCCGGGTCAAT

MKI67	Sequence (5′ → 3′)
Forward Primer	ACGCCTGGTTACTATCAAAAGG
Reverse Primer	CAGACCCATTTACTTGTGTTGGA

TYMS	Sequence (5′ → 3′)
Forward Primer	CTGCTGACAACCAAACGTGTG
Reverse Primer	GCATCCCAGATTTTCACTCCCTT

LYPD3	Sequence (5′ → 3′)
Forward Primer	GATGCTCCCCGAACAAGATGA
Reverse Primer	CAGCGAGAATTGTCCGTGGAT

### 2.15 Experiment of Cell Counting Kit‐8 (CCK‐8)

The CCK‐8 assay was conducted employing the solution sourced from Dojindo Laboratories Inc. Throughout the procedure, rigorous adherence to the manufacturer's guideline. The cells were planted in 96‐well plates, maintaining a concentration of 2 × 10^3^ cells per 100 μL in each well. Postincubation intervals of 0, 24, 48, 72 or 96 h, CCK‐8 solution was introduced into the 96‐well plates at a concentration of 10 μL per well, followed by a 2‐h cell viability assessment postincubation, and the 450 nm absorbance was analysed using a microplate reader.

### 2.16 Colony Formation

In the colony formation assay, LUAD cells were seeded into six‐well plates at a density of 1 × 10^3^ cells per well to assess their clonogenic potential. Subsequently, the plates were incubated under optimal conditions of 37°C and 5% CO_2_ for a duration of 14 days, with regular replenishment of the culture medium every 4 days to ensure cell viability and growth. Upon completion of the incubation period, the colonies were fixed in place using 4% paraformaldehyde and subsequently stained with 0.1% crystal violet, sourced from the reputable Beyotime Institute of Biotechnology. In every well, the colony count was conducted, ensuring each colony contained over 50 cells. The experiments were conducted in three separate, identical repetitions.

### 2.17 Transwell Assay

Chambers of Transwell (with pore sizes of 8‐μm; produced by Corning Inc.) were utilised in assessing the migratory abilities of LUAD cells. Within every upper section of the Transwell chamber, 300 μL of serum‐free medium was used to seed 4 × 10^4^ cells, and the lower section was occupied with 700 μL of DMEM medium enriched with 10% FBS. After a two‐day incubation period, the cells underwent a fixation process using 4% paraformaldehyde and subsequently were stained with 0.1% crystal violet, procured from the Beyotime Institute of Biotechnology. To ensure accuracy, nonmigratory cells adhering to the upper chamber membrane of the insert were meticulously removed with a cotton swab. Subsequently, images of the stained cells were captured utilising an inverted microscope. For quantitative assessment of the migratory potential of LUAD cells, cell counts were systematically performed in five randomly designated microscopic fields. The entire experimental procedure was repeated in triplicate.

### 2.18 Wound‐Healing Assay

When the concentration of LUAD cells reached 90%, they were plated onto six‐well dishes. Subsequently, a linear scratch wound was created within the monolayer of cells using a 20‐μL pipette tip. A precise linear scratch wound was delicately introduced into the confluent monolayer of cells, employing a 20‐μL pipette tip as the tool of choice. Following this manipulation, the cells were cultured in a serum‐free medium, followed by meticulous washing with PBS to eliminate debris and resuspend the cells. Images of the wounded areas were then captured at 0 and 24 h utilising an inverted microscope.

### 2.19 Statistical Analysis

The initial processing of data, encompassing statistical analysis and visualisation of the outcomes, was carried out utilising the R software, specifically version 4.3.0, in conjunction with the Perl software version 5.30.0. To assess the relationship between continuous variables, Pearson and Spearman correlation coefficients were computed. Categorical data, on the other hand, were statistically examined through the utilisation of the Chi‐square test for comparison, while the comparison was performed by the use of either the Wilcoxon rank‐sum test or *t*‐test when involved in continuous variables. All statistical analyses were performed adopting a two‐sided approach, a *p* value threshold of less than 0.05 was rigorously applied to determine statistical significance.

## 3 Results

### 3.1 A scRNA‐Seq Approach to Cellular Heterogeneity and PCD Dynamics

**FIGURE 1:**
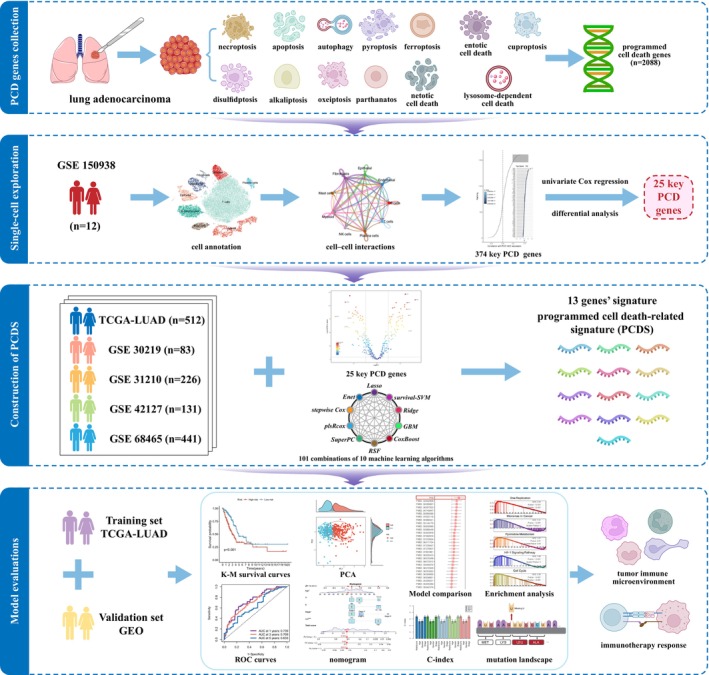
Flow chart of this study.

**FIGURE 2:**
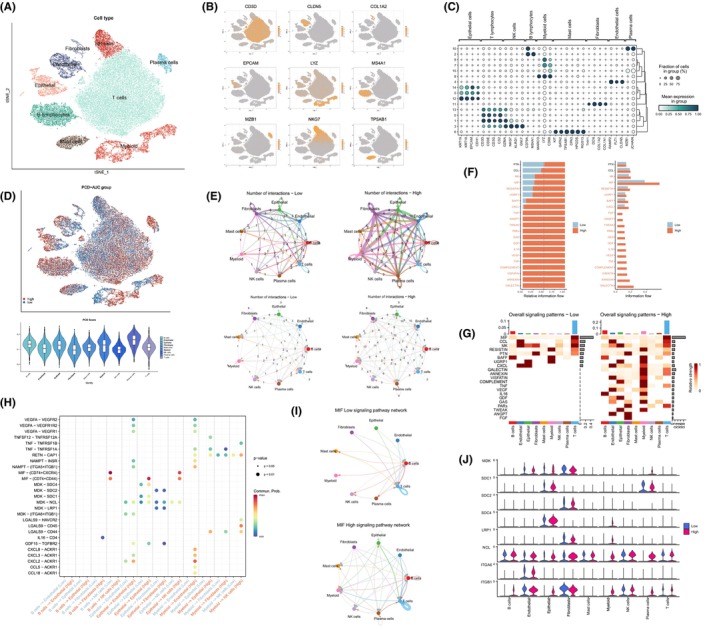
A scRNA‐seq approach to cellular heterogeneity and PCD dynamics. (A) Cell annotation revealed 9 major cell types. (B) The t‐SNE plot displayed the expression distribution of typical marker genes of different cell types in LUAD samples. (C) The association between typical marker genes of different cell types in LUAD and the 16 clusters was visually represented through the bubble plot. (D) The t‐SNE plot showed the cell distribution of the high and low PCD‐AUC groups. The violin plot illustrates the PCD activity scores of cells across different cell types. (E) The network plot compared the cell communication quantity and cell communication intensity between the high and low PCD‐AUC groups. (F) Identification and visualisation of signalling pathways in the high and low PCD‐AUC groups. (G) Comparison of signalling pathway strength between the high and low PCD‐AUC groups through a heatmap. (H) Identification of upregulated and downregulated signalling ligand‐receptor pairs in different cell subgroups of the high and low PCD‐AUC groups. (I) Differences in cell communication in the MIF pathway between the high and low PCD‐AUC groups. (J) Expression patterns of ligand‐receptor pairs in the MIF pathway in different cell populations.

The workflow diagram of this study is illustrated in Figure 1. A Univariate Cox regression study was performed on the transcriptomic and clinical information from the TCGA‐LUAD data set concerning these 2088 genes associated with PCD (Table S1). The study pinpointed 455 genes linked to PCD, each with notable predictive significance (*p* < 0.05). The processing of scRNA‐seq data from 12 LUAD patients was beginning with cell quality control, and a total of 45,761 cells of high quality were screened for subsequent analysis (Figure S1A). The ‘Harmony’ package was employed to eliminate batch discrepancies among samples, resulting in relatively consistent cell distributions across the 12 samples (Figure S1B). Subsequently, dimensionality reduction was performed using PCA and t‐SNE functions, resulting in the classification of all cells into 16 clusters through cluster analysis (Figure S1C). Following annotation, nine major clusters were generated, encompassing T cells, NK cells, B cells, mast cells, epithelial cells, endothelial cells, fibroblasts, myeloid cells and plasma cells (Figure 2A). The t‐SNE plot and bubble plot demonstrate the expression distribution of representative marker genes for different cell types and their associations with each cell cluster (Figure 2B,C). The proportion plot illustrates the distribution differences of cell types among the 12 LUAD samples (Figure S1D). Based on the screened 455 PCD‐related genes, we utilised the AddModuleScore and AUCell algorithms to assess the PCD activity of cells across nine different cell types. The results indicated that plasma cells exhibited the highest PCD activity, followed by B cells, while fibroblasts showed the lowest PCD activity (Figures 2D and S1E). This suggests that PCD may play an irreplaceable role in specific immune responses, potentially involving processes such as antigen presentation, immune regulation and antibody production. Following this, each cell was classified into groups with high and low PCD‐AUC, based on their median AUC scores (Figure 2D).

### 3.2 Cell–Cell Interactions

The cell communication between the two groups was evaluated using cellchat analysis. Compared to the group with low PCD‐AUC, the high PCD‐AUC group demonstrated increased cell communication in both quantity and intensity (Figures 2E and S2A). We found that the high PCD‐AUC group exhibited a general enhancement in the strength of both incoming and outgoing interactions, such as an observed significant increase in the strength of incoming interactions in B cells and myeloid cells (Figure S2B). Additionally, the higher expression levels of most signalling pathways were observed in the high PCD‐AUC group (Figure 2F,G). The bubble plot reveals that cells in the high PCD‐AUC group exhibit enhanced intercellular communication through a greater number of signalling ligands. Particularly, in the interactions between epithelial cells, myeloid cells and endothelial cells, ligand pairs such as CXCL2‐ACKR1 and CXCL8‐ACKR1 are upregulated (Figure 2H). Macrophage Migration Inhibitory Factor (MIF) appears to play a crucial role in both the high and low PCD‐AUC groups. (Figures 2F,G and S2C,D). MIF is well‐known for its involvement in various biological processes including inflammation, immune regulation, cell proliferation and survival, with potential to be associated with tumour immune evasion and tumour progression [44, 45]. Significant upregulation of the MIF pathway and MIF‐related genes, including CD74, CXCR4 and CD44, was observed in the high PCD‐AUC group (Figure 2I,J). This finding suggested the potential of MIF (CD74 + CXCR4) axis to serve as a key signalling pathway in promoting the interactions between different cell populations within the high PCD‐AUC group. In addition, a significant upregulation of the Chemokine (C‐C motif) Ligand family (CCL) and the Megakaryocyte (MK) pathway was observed in the high PCD‐AUC group (Figure S2E,F).

### 3.3 Developing a Predictive Signature Utilising Integrative Machine Learning Techniques

**FIGURE 3:**
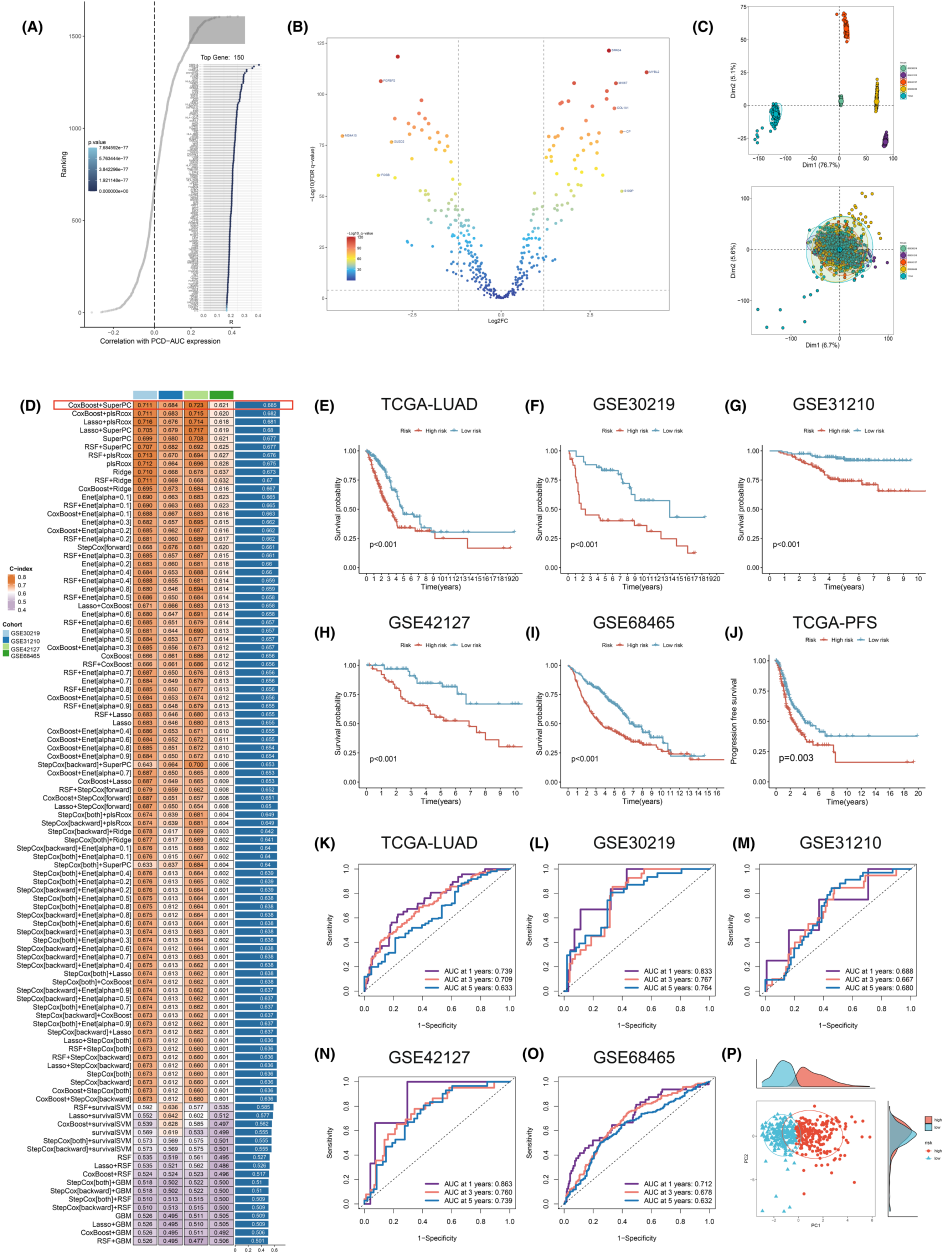
Construction and evaluation of the PCDS. (A) The top 150 genes with the highest correlations to PCD. (B) The volcano plot identified and visualised a total of 145 differentially expressed genes (DEGs) (*p* < 0.05, |log2FC| > 1.5). (C) The PCA plot demonstrated the sample distribution before and after batch correction in five datasets used for model building. (D) A total of 101 prediction models were established using a machine learning computational framework, and the C‐index of each model was calculated across all validation datasets. (E–I) (K–M) curves of OS were generated based on PCDS in TCGA‐LUAD (*p* < 0.001) (E), GSE30219 (*p* < 0.001) (F), GSE31210 (*p* < 0.001) (G), GSE42127 (*p* = 0.001) (H), and GSE68465 (*p* < 0.001) (I). (J) The PCDS was further evaluated using (K–M) curves of PFS in TCGA‐LUAD (*p* < 0.05). (K–O) Time‐dependent ROC curves based on PCDS were plotted for TCGA‐LUAD (K), GSE30219 (L), GSE31210 (M), GSE42127 (N), and GSE68465 (O). (P) The distribution of patients from the high‐risk and low‐risk groups in the TCGA‐LUAD dataset was visualised in the PCA plot.

Through the amalgamation of genes with varying expression levels (DEGs, |log2FC| > 0.7, *p* < 0.05) among groups with high and low PCD‐AUC and the leading 150 genes exhibiting the strongest correlation with PCD, we identified a total of 374 genes (Figure 3A, Tables S2 and S3). Transcriptomic data for these genes were obtained from the TCGA and GTEx databases, and batch effects were removed (Figure S3A,B). Subsequent differential analysis identified 145 DEGs associated with PCD (*p* < 0.05, |log2FC| > 1.5) (Figures 3B and S3C, Table S4). The TCGA‐LUAD data set's transcriptomic and clinical information were utilised to conduct a univariate Cox regression analysis. The study pinpointed 38 genes linked to PCD that demonstrated notable predictive significance (*p* < 0.05). The TCGA‐LUAD data set was employed as the training data set, whereas GSE30219, GSE31210, GSE42127 and GSE68465 functioned as the validation sets, and batch effects were eliminated (Figure 3C). Due to gene loss during the batch removal process, a final model was constructed using 25 key genes (Table S5). In order to develop a consensus PCDS, a total of 101 ML algorithms were combined and utilised in the analyses of 25 key genes. As a result, a total of 101 models were constructed using different ML algorithms. Subsequently, the C‐index of each model among all validation data sets was calculated (Figure 3D). Notably, the best‐performing model, which combined CoxBoost and SuperPC, exhibited the highest averaged C‐index (0.685). Then, 13 key genes were identified utilising the algorithm, including MKI67, PTX3, TYMS, S100P, LAD1, LYPD3, SCNN1B, IL1A, SPIB, SLC34A2, CHPF, IER3 and CD79A. Subsequent to the identification of these 13 genes, a tailored algorithm was employed to compute individualised risk scores for each patient sample, patients were subsequently stratified into distinct cohorts: those classified as high risk and those designated as low risk according to their median risk scores.

### 3.4 Evaluations of PCDS

**FIGURE 4:**
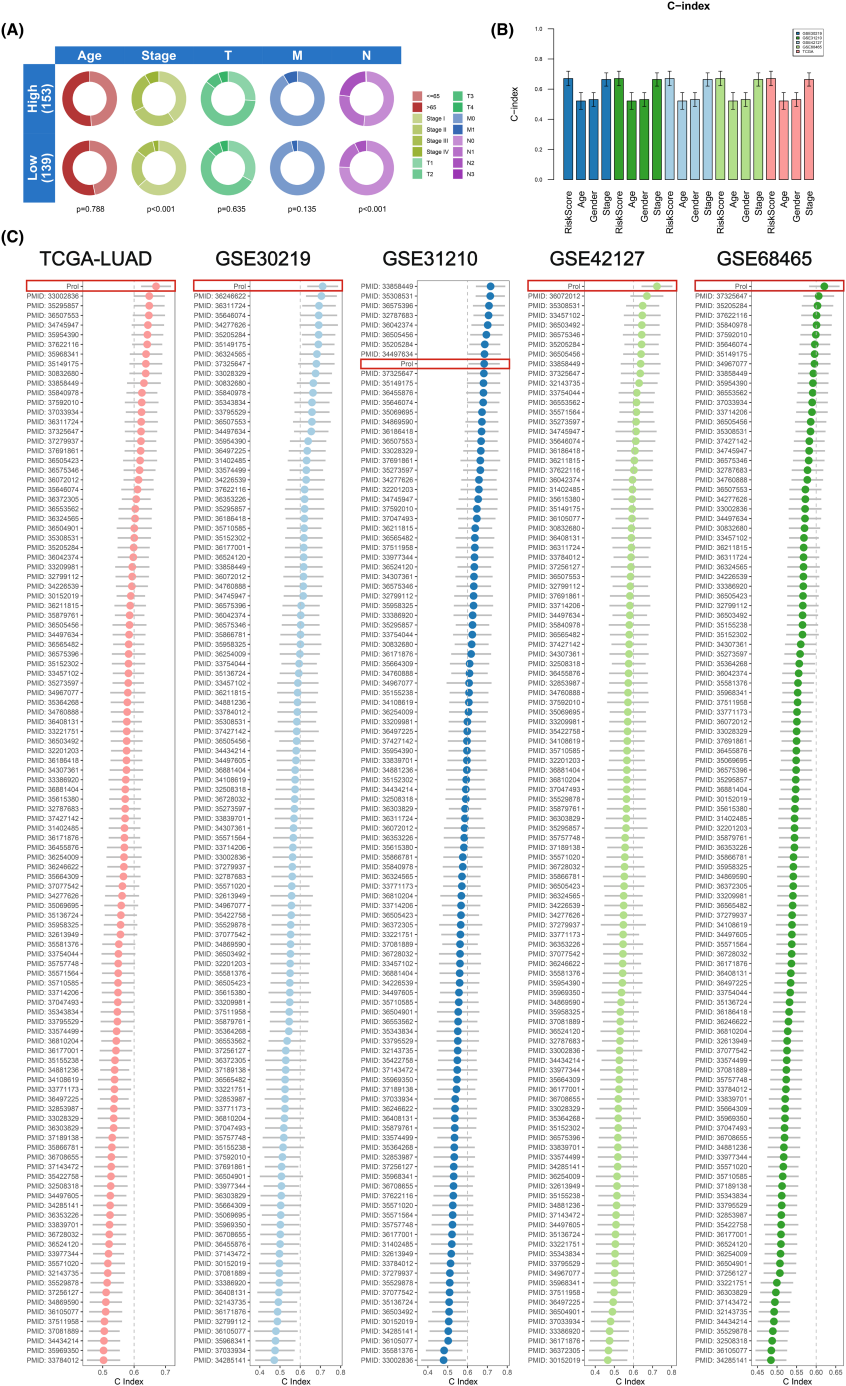
Prognostic performance of the PCDS. (A) The clinical correlation circle plot displayed the correlation of risk score with age, stage, T‐stage, N‐stage, and M‐stage in the TCGA‐LUAD dataset. (B) The bar graph visually presented the C‐index of the risk score and clinical features such as age, gender, and stage in the TCGA, GSE30219, GSE31210, GSE42127, and GSE68465 datasets. (C) The C‐index of PCDS and 140 previously published LUAD prognostic models in the TCGA, GSE30219, GSE31210, GSE42127, and GSE68465 datasets was calculated and compared.

Between high‐ and low‐risk groups, further analysis was conducted on risk score distribution, duration of patient survival and overall patient survival condition, accompanied by an analysis of the expression trends of the chosen 13 modelling genes. The findings from the analysis of these five data sets unveiled, patients belonging to the high‐risk group consistently exhibited elevated risk scores, which were associated with notably shorter survival durations and a heightened likelihood of mortality. Additionally, the expression levels of MKI67, PTX3, TYMS, S100P, LAD1, LYPD3, IL1A, CHPF and IER3 displayed a significant positive correlation with the risk scores, indicating their potential as prognostic risk factors (Figure S4). Subsequently, separate survival analyses were performed for the high‐risk and low‐risk groups in these five data sets, generating Kaplan–Meier survival curves. The OS rate for the group at high risk was notably less than the low‐risk group (*p* ≤ 0.001, Figure 3E–I). Additionally, within the training data set, the group at high risk showed a lower rate of progression‐free survival (PFS) (*p* = 0.003, Figure 3J). Assessing PCDS's forecasting accuracy in LUAD involved charting ROC curves over time and computing AUC values for every data set. The AUC figures for the 1‐, 3‐ and 5‐year intervals were listed below: 0.739, 0.709, 0.633 (TCGA‐LUAD); 0.833, 0.767, 0.764 (GSE30219); 0.688, 0.667, 0.680 (GSE31210); 0.863, 0.760, 0.739 (GSE42127); 0.712, 0.678, 0.632 (GSE68465) (Figure 3K–O). These results demonstrated the strong predictive performance of PCDS in prognosticating LUAD patients. Subsequently, PCA was performed for dimensionality reduction and visualisation to further observe the differences in sample distribution between the two groups. The results consistently revealed significant differences in sample distribution between the two groups (Figures 3P and S5A). Additionally, in the ROC curves combined with clinical characteristics, the AUC values for risk and clinical factors such as age, gender and stage were as follows: 0.709, 0.533, 0.517, 0.680 (TCGA‐LUAD); 0.767, 0.523, 0.525, 0.590 (GSE30219); 0.667, 0.634, 0.602, 0.742 (GSE31210); 0.760, 0.682, 0.565, 0.631 (GSE42127); 0.678, 0.550, 0.576, 0.676 (GSE68465) (Figure S5B). Compared with other clinical features in the TCGA‐LUAD, GSE30219, GSE42127 and GSE68465 data sets, PCDS demonstrated superior predictive performance. Furthermore, our research findings demonstrated a favourable association between PCDS and the N stage and clinical stage in the TCGA‐LUAD data set (*p* < 0.05) (Figure 4A). Additionally, PCDS exhibited the highest C‐index values across the five data sets (Figure 4B).

### 3.5 Comparison of Signatures

In the past few tens of years, significant advancements in advanced sequencing and bioinformatics technologies have accelerated the development of ML‐based signatures for predicting the prognosis of LUAD patients. To assess the predictive capabilities of PCDS in more detail, we conducted a comprehensive search on PubMed and retrieved 140 published signatures, including mRNA and noncoding RNA signatures associated with various biological processes such as cellular immunity, aging, metabolism, autophagy, ferroptosis, m6A RNA methylation and inflammation. Subsequently, we compared the predictive performance of PCDS and other signatures by examining the C‐index (Figure 4C) in the TCGA, GSE30219, GSE31210, GSE42127 and GSE68465 datasets. Consistent results demonstrated that the C‐index values of PCDS ranked first in the TCGA, GSE30219, GSE42127 and GSE68465 data sets, and ninth in the GSE31210 data set. This indicates that PCDS outperforms other signatures in terms of predictive performance.

### 3.6 The Development and Validation of a Nomogram

**FIGURE 5:**
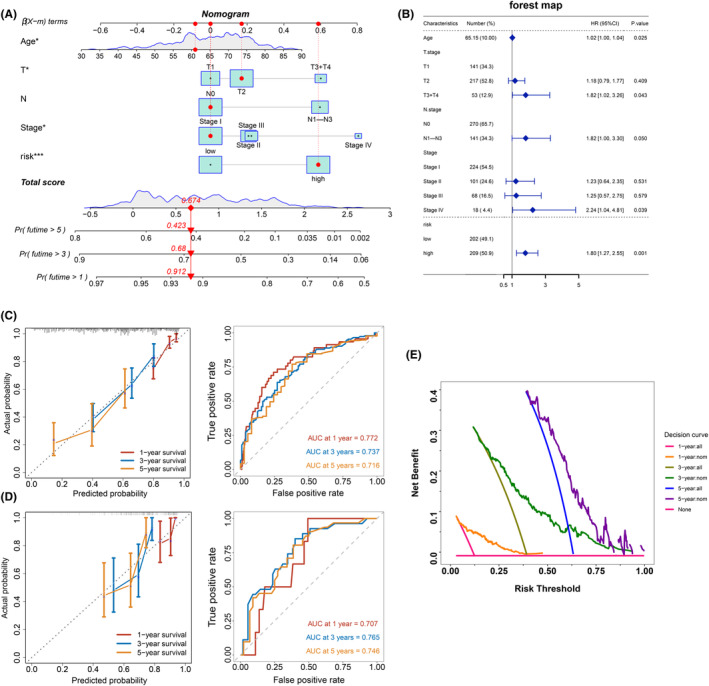
Establishment and validation of a nomogram combined with clinical characteristics. (A) In TCGA‐LUAD, a Nomogram was constructed to predict 1‐, 3‐, and 5‐year survival of LUAD patients by integrating PCDS, age, and TNM staging. (B) The forest plot displayed age, T stage, N stage, staging, and PCDS risk score as key prognostic factors in the Nomogram. (C) Calibration curves and time‐dependent ROC curves of the Nomogram were plotted in the training set (TCGA‐LUAD). (D) Calibration curves and time‐dependent ROC curves of the Nomogram were plotted in the test set (GSE30219). (E) The decision curve analysis (DCA) curve of the Nomogram for survival prediction was conducted in the training set (TCGA‐LUAD).

The TNM staging system is widely used in the global practice of LUAD diagnosis and treatment. Therefore, we have combined TNM staging with PCDS to enhance prognostic prediction performance. Subsequently, using this nomogram, we calculated scores for each patient, thereby achieving more accurate prognostic assessment (Figure 5A). Furthermore, we conducted Cox analysis based on this nomogram and identified age, T stage, N stage, staging and PCDS risk score as key prognostic factors (*p* < 0.05, Figure 5B). We assessed the predictive accuracy of our nomogram in both the training and testing sets. Specifically, we chose the GSE30219 data set as the validation testing set. Calibration curves (Figure 5C,D) effectively illustrated the alignment between the predicted values and the observed outcomes. Furthermore, we conducted time‐dependent ROC analysis using this nomogram, both in the training set (TCGA‐LUAD) and the testing set (GSE30219). The resulting AUC values for 1‐, 3‐ and 5‐year were as follows: 0.772, 0.737, 0.716 (TCGA‐LUAD); 0.707, 0.765, 0.746 (GSE30219). All AUC values exceeded 0.7, indicating the robust prognostic predictive performance of our nomogram in LUAD patients (Figure 5C,D). Lastly, to gauge the practical usefulness and efficacy of our nomogram, we employed decision curve analysis (DCA). This analysis highlighted the potential of our nomogram to precisely forecast the survival probability of LUAD patients across various time intervals (Figure 5E).

### 3.7 Underlying Biological Mechanisms

**FIGURE 6:**
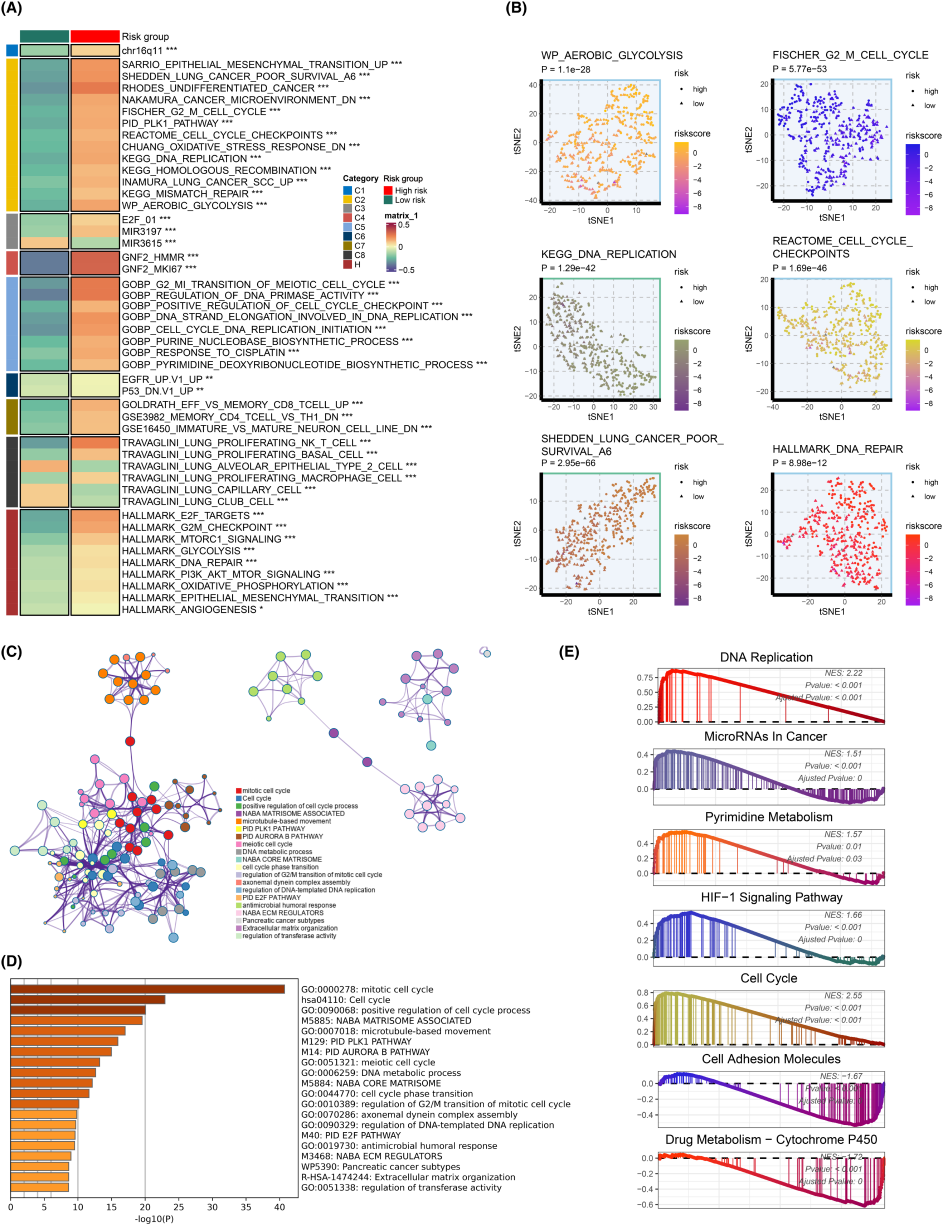
Underlying biological mechanisms associated with PCDS. (A) GSVA analysis depicted the biological features of high and low‐risk groups. (B) The T‐SNE plot illustrated the differences in pathway activity between the high‐risk and low‐risk groups. (C, D) The network plot (C) and bar graph (D) displayed the enrichment analysis of differentially expressed genes (DEGs) between the high‐risk and low‐risk groups performed using Metascape. (E) GSEA analysis was conducted for the gene ontology (GO) and Kyoto Encyclopedia of Genes and Genomes (KEGG) terms specifically associated with PCDS.

PCD, plays a pivotal role in numerous crucial cellular occurrences, including the intricate immune response, metabolic regulation and cell signalling transduction [7, 10]. These events hold a pivotal position in the emergence and subsequent progression of lung adenocarcinoma. Therefore, our aim is to reveal the intricate biological mechanisms that underlie and govern PCD and its correlation with the tumour biology processes in lung adenocarcinoma. The results of Gene Set Variation Analysis (GSVA) demonstrate a noteworthy positive correlation has been observed between the risk score derived from PCDS and multiple cancer‐promoting pathways, along with epithelial‐mesenchymal transition (EMT), cell cycle regulation, oxidative stress response, DNA replication, glycolysis, and PI3K‐AKT–mTOR signalling (Figure 6A). Furthermore, we have also observed the association of PCDS with prognosis in lung cancer, TME and response to chemotherapy drug cisplatin. Subsequently, we calculated the enrichment scores of pathways for each sample and reduced them to a two‐dimensional plane, revealing that samples can be differentiated based on the risk score of PCDS (Figure 6B). Metascape analysis indicates that DEGs between the two risk groups are enriched in pathways related to cell cycle regulation, DNA metabolism and DNA replication (Figure 6C,D). Moreover, the Gene Set Enrichment Analysis (GSEA) results demonstrate that the high‐risk group is enriched in pathways associated with DNA replication, tumour‐related microRNAs, pyrimidine metabolism, HIF‐1 signalling pathway and cell cycle, while the low‐risk group exhibits a concentration of pathways related to cell adhesion molecules and drug metabolism—cytochrome P450, which in line with our previous research outcomes (Figure 6E).

### 3.8 Genomic Alterations

**FIGURE 7:**
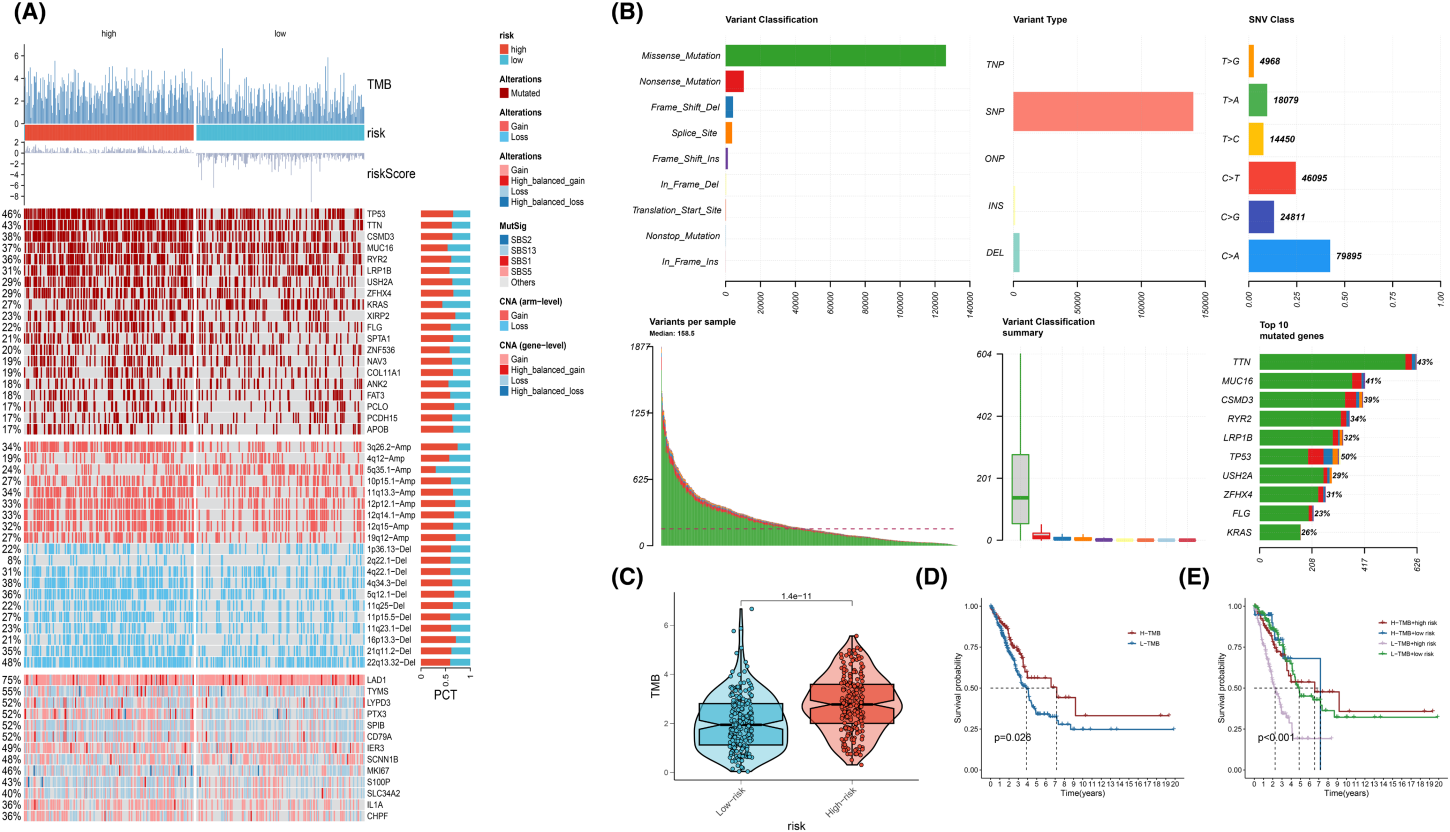
The Genomic variation landscape between high and low‐risk groups. (A) The heatmap compared the genomic landscape of the high‐risk and low‐risk groups. (B) Visualisation represented the details of mutations. (C) The violin plot depicted the differences in tumour mutational burden (TMB) between the high‐risk and low‐risk groups. (D, E) Survival analysis was performed for LUAD patients based on TMB (D) and the integration of risk scores and TMB (E).

Genomic variations and TMB is of critical importance in the analysis of tumour bioinformatics, aiding in the establishment of molecular subtypes, prediction of immune therapy response and patient prognosis, as well as identification of tumour driver genes for precision medicine and targeted treatment guidance [46, 47]. Through our analysis, we successfully identified the top 20 genes exhibiting the highest mutation frequencies were identified in both the high‐risk and low‐risk groups. In these groups, genes such as TP53, TTN, CSMD3, MUC16, RYR2 and LRP1B exhibited a broader range of mutations, particularly in the high‐risk group. Additionally, the high‐risk group pronounced elevation in chromosomal instability (CIN) was observed (Figure 7A). Among the mutations identified, missense mutations stood out as the most prevalent type, and the incidence of C>A mutations was higher relative to C>T and C>G transitions (Figure 7B). Subsequently, we calculated the TMB scores for both risk groups and found significantly higher scores in the high‐risk group (Figure 7C, *p* < 0.05). To investigate the impact of TMB on prognosis, all LUAD patients were divided into two subsets based on high TMB and low TMB. The Kaplan–Meier analysis highlighted a positive association between elevated TMB levels and improved overall survival (Figure 7D, *p* < 0.05). To further enhance the prognostic prediction for LUAD patients, we conducted additional analyses that incorporated both TMB scores and risk scores. These analyses resulted in the categorisation of patients into four distinct groups, based on the median values of both the risk score and TMB score. The survival analysis illuminated a pivotal insight: patients with high tumour mutation burden (H‐TMB) coupled with a low‐risk score demonstrated the most favourable prognosis, conversely, the group characterised by low tumour mutation burden (L‐TMB) and a high‐risk score fared the worst, thereby emphasising the enhanced prognostic value achieved through the combination of these two indicators (Figure 7E, *p* < 0.05).

### 3.9 Immune Infiltration

**FIGURE 8:**
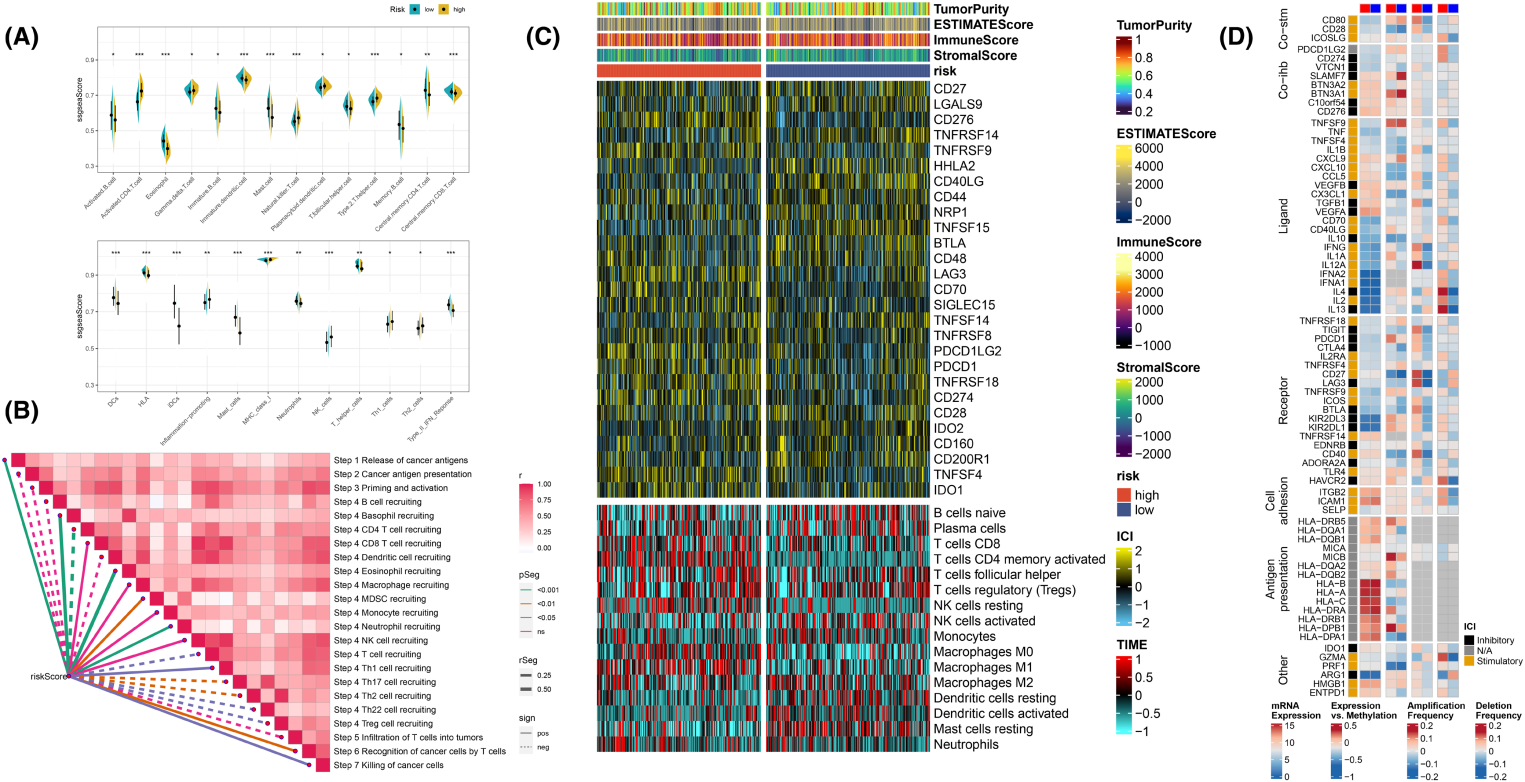
The correlation between PCDS and immune infiltration characteristics. (A) The ssGSEA algorithm assessed the differences in immune cell infiltration abundance and immune function between the high‐risk and low‐risk groups. (B) The correlation between PCDS scores and key steps in the cancer‐immunity cycle. (C) The heatmap compared the differences between the high‐risk and low‐risk groups in terms of immune checkpoint expression and immune cell infiltration. (D) The correlation between PCDS and immune modulators.

The tumour immune microenvironment (TIME) is intimately linked to tumour development, occupying a pivotal position in initial phases and the metastatic process, including immune surveillance, immune regulation and tumour immune evasion [48, 49]. To explore the immune status reflected by the PCDS, we conducted an analysis investigating the relationship between PCDS and immune cells, immune checkpoints (ICs), cancer immune cycle and immune modulators. Firstly, the ESTIMATE algorithm was employed to compute the immune score, stromal score, and ESTIMATE score for each individual, followed by a comparative analysis of immune cell infiltration alterations between the two distinct risk groups. The outcomes of this investigation revealed an inverse relationship between the risk score and the immune score, stromal score, as well as the ESTIMATE score, concurrently exhibiting a direct correlation with tumour purity. This indicates that patients harbouring low‐risk scores potentially possess a more inflamed tumour immune microenvironment (Figure S6A). The immune microenvironment of the two risk groups was evaluated using the ssGSEA algorithm. The research findings revealed that the low‐risk group exhibited higher ssGSEA scores in many immune cell types, including activated B cells, eosinophils, immature B cells, immature dendritic cells, mast cells, helper T cells, follicular helper T cells, memory B cells, central memory CD4 T cells, central memory CD8 T cells and neutrophils (Figure 8A, *p* < 0.05). Moreover, the ssGSEA scores for human leukocyte antigen (HLA) and type II interferon response were also elevated in the low‐risk group (Figure 8A, *p* < 0.05). Thereafter, to ensure the robustness of our evaluations, seven distinct algorithms were employed to scrutinise the interplay between the risk score and the abundance of tumour‐infiltrating immune cells. The findings of this investigation revealed an inverse relationship between the risk score and the infiltration rates of diverse immune cell subsets, notably CD8^+^ T cells, B cells, NK cells and mast cells [42, 43]. This observation suggests increased immune cell infiltration in the TME of the low‐risk group (Figure S6B,C). The PCDS score showed a negative correlation with key aspects of the cancer immune cycle, including the presentation of cancer antigen priming and activation, the recruitment of B cells, and the recruitment of CD4^+^ T cells, dendritic cell recruiting, Th17 cell recruiting, Th2 cell recruiting, Th22 cell recruiting, Treg cell recruiting, and T cell infiltration into the tumour stroma (Figure 8B). Furthermore, we found increased expression levels of ICs such as CD27, CD28, CD44, CD48, CD160, CD200R1, CD40LG, TNFSF14, TNFRSF14, TNFSF15, BTLA and LGALS9 in patients with low‐risk scores (Figure 8C). This implies that patients with low risk scores may be more likely to benefit from immune therapies targeting these specific ICs. Finally, we watched elevated levels of immune modulators in the low‐risk group (Figure 8D). In summary, the low‐risk group exhibited higher levels of immune cell infiltration, immune checkpoint expression and immune modulator enrichment, indicating a relatively immune‐promoting inflammatory microenvironment that may serve as potential beneficiaries of immune therapy.

### 3.10 Response to Immunotherapy and Drug Sensitivity

**FIGURE 9:**
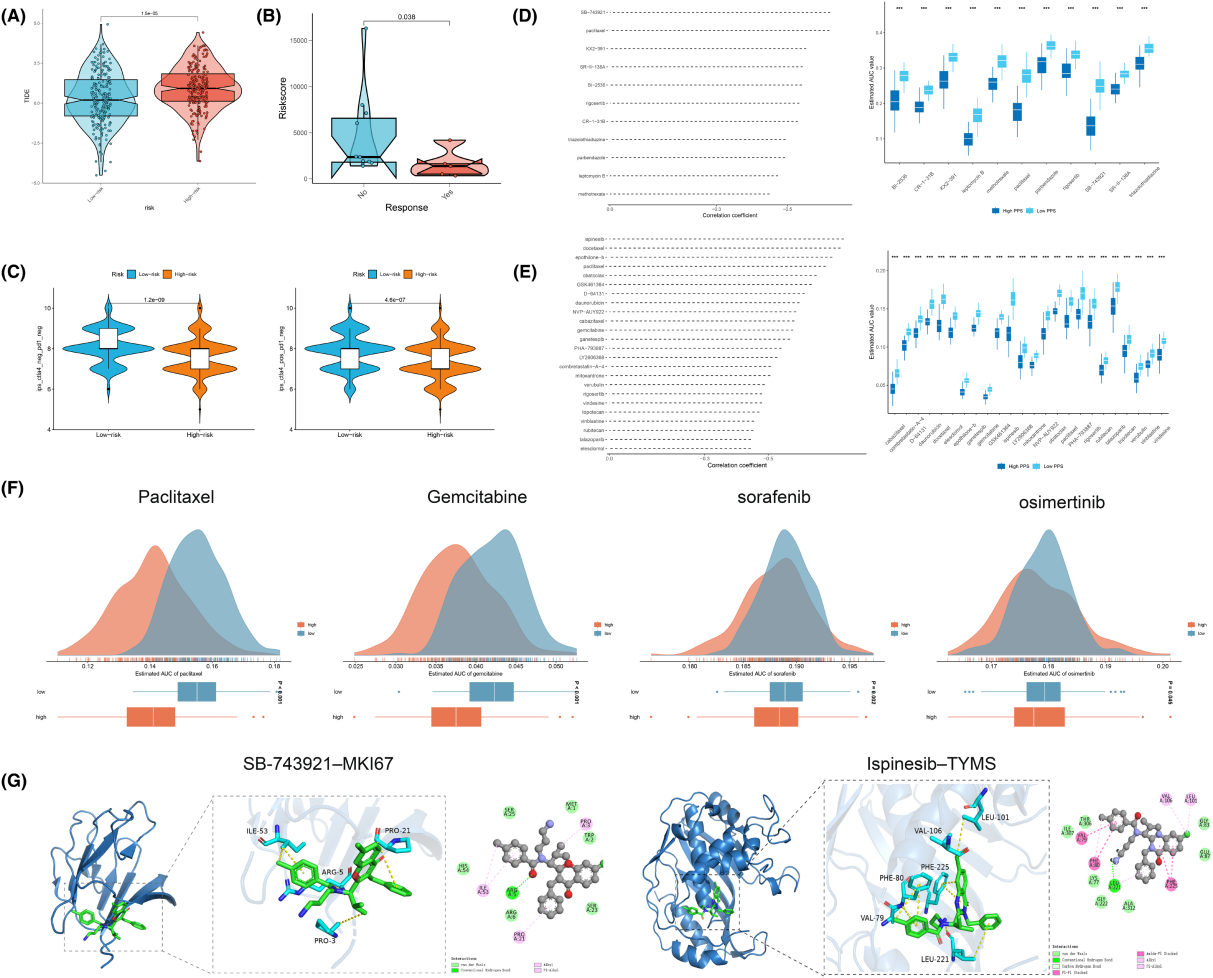
Estimation of immunotherapy response and drug sensitivity. (A) The violin plots compare the TIDE scores between the high and low‐risk groups. (B) The association between PCDS risk scores in the GSE126044 dataset and immune therapy response. (C) Comparing the IPS scores between the high and low‐risk groups and predicting their potential response to ICB drugs. (D, E) Predictive models of drug resistance based on the CTRP2.0 (D) and PRISM (E) datasets yielded a total of 11 CTRP derivatives and 24 PRISM derivatives. (F) Using the PRISM database, the sensitivity of the high and low‐risk groups to four commonly used clinical drugs (Paclitaxel, gemcitabine, sorafenib, and osimertinib) was compared. (G) Molecular docking models depict the interactions between the MKI67 protein and SB‐743921, as well as the interactions between the TYMS protein and ispinesib.

Recently, the comprehensive treatment of advanced tumours increasingly relies on immune checkpoint blockade (ICB) drugs targeting PD‐1/PD‐L1 and CTLA‐4 [50]. To further explore the potential role of PCDS in predicting immune therapy response, the correlation between tumour immune evasion and PCDS risk scores was evaluated using the TIDE website. Individuals belonging to the high‐risk group may exhibit a heightened tendency towards immune evasion, which could result in suboptimal response to immune therapy (Figure 9A, *p* < 0.05). Subsequently, the GSE126044 data set, which includes transcriptomic data of 16 non‐small cell lung cancer patients treated with anti‐PD‐1 drugs, including responders and non‐responders, was utilised. PCDS risk scores were calculated based on their transcriptomic data and divided into high‐ and low‐risk groups. It was observed that patients with low‐risk scores exhibited more favourable response to immune therapy (Figure 9B, *p* < 0.05). Furthermore, the Immune Prognostic Score (IPS) of LUAD patients was obtained from the TCIA database to assess their immunogenicity and predict potential response to ICB drugs. It was found that the low‐risk group had elevated IPS scores, indicating that this group may have a more favourable response to ICB (Figure 9C, *p* < 0.05). In conclusion, the low‐risk group demonstrated a stronger immune response, making them more prone to experiencing positive outcomes from immune therapy and potentially leading to better prognostic outcomes.

Considering that chemotherapy combined with targeted therapy remains the standard treatment for advanced non‐small‐cell lung cancer [51], the CTRP2.0 and PRISM databases were employed to predict potentially effective small‐molecule drugs aimed at improving treatment response and prognosis in the high‐risk group. Based on the Spearman correlation (Spearman correlation coefficient *r* < −0.35, *p* < 0.05) between risk scores and AUC scores, 11 CTRP2.0 derivatives (including SB‐743921, paclitaxel, etc.) and 24 PRISM derivatives (including ispinesib and docetaxel) were identified. It was found that all 28 derivatives had lower AUC values in the high‐risk group (Figure 9D,E). Paclitaxel, gemcitabine, sorafenib and osimertinib are four commonly used clinical drugs, and using the PRISM database, it was discovered that high‐risk patients exhibited stronger sensitivity to these four drugs (Figure 9F). Subsequently, SB‐743921 and ispinesib were selected as two drugs, and molecular docking was carried out utilising software such as MOE 2019 to assess the interaction with proteins encoded by MKI67 and TYMS (two PCDS modelling genes) (Figures 9G and S7). The docking scores between the proteins and SB‐743921, ispinesib were as follows: −6.6061 kcal/mol (MKI67‐SB‐743921), −6.2455 kcal/mol (MKI67‐ispinesib); −6.9090 kcal/mol (TYMS‐SB‐743921), −6.8666 kcal/mol (TYMS‐ispinesib). The low binding energies in the molecular docking indicate strong binding affinity between the compounds and the protein targets. The docking scores between these two drugs and the two modelling genes were all below −6 kcal/mol, indicating a perfect interaction between the drugs and the modelling genes. To validate these findings, the GDSC database was further utilised to calculate the IC50 values of various commonly used antitumor drugs. The results showed significantly higher IC50 values for chemotherapy drugs (such as 5‐fluorouracil, cyclophosphamide, cytarabine, oxaliplatin) and targeted therapy drugs (such as crizotinib, erlotinib and gefitinib) in the high‐risk group. This finding suggests that patients with high‐risk scores may have a better response to these drugs (Figure S8), which could potentially compensate for the unfavourable response to immunotherapy in the high‐risk group.

### 3.11 Verification of MKI67 Expression and Its Biological Role in LUAD

**FIGURE 10:**
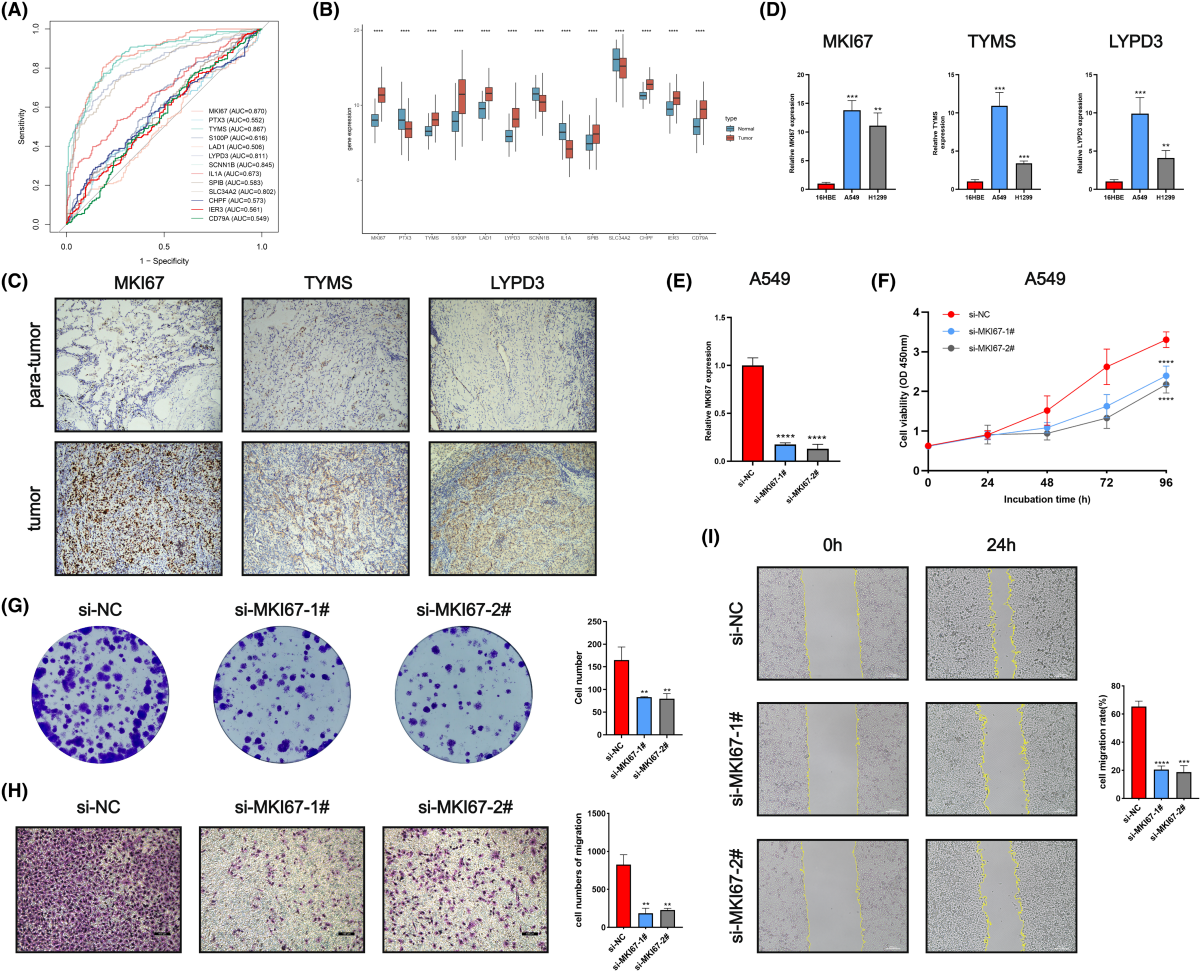
Validation of MKI67 expression and biological function in LUAD. (A) The ROC curve demonstrated the correlation between 13 modelling genes and the PCDS. (B) Differential expression of the 13 modelling genes in LUAD and normal lung tissues was compared using the TCGA and GTEx databases. (C) Immunohistochemistry experiments revealed high protein expression of MKI67, TYMS, and LYPD3 in LUAD compared to para‐tumour tissues. (D) Expression levels of MKI67, TYMS, and LYPD3 were assessed using RT‐qPCR in 16HBE, H1299, and A549 cells. (E) The knockdown efficiency of MKI67 in the A549 cell line was validated using RT‐qPCR. The impact of MKI67 knockdown on LUAD cell proliferation was determined using the CCK‐8 assay (F) and colony formation assay (G). The effect of MKI67 knockdown on LUAD cell migration was explored using the Transwell assay (H) and wound healing assay (I).

We evaluated the correlation between 13 key genes and the PCDS using ROC curves. We identified the top five genes most closely associated with the PCDS, namely MKI67 (AUC = 0.870), TYMS (AUC = 0.867), SCNN1B (AUC = 0.845), LYPD3 (AUC = 0.811) and SLC34A2 (AUC = 0.802) (Figure 10A). Utilising transcriptomic data from the TCGA and GTEx databases, we conducted an assessment of the differential expression of these 13 modelling genes in LUAD as compared to normal lung tissues. The findings revealed that MKI67, TYMS, and LYPD3 were relatively highly expressed in LUAD, while SCNN1B and SLC34A2 were relatively lowly expressed (Figure 10B). These findings were further validated using immunohistochemistry (IHC) images obtained from the Human Protein Atlas (HPA) database (Figure S9). Furthermore, immunohistochemistry experiments performed on LUAD and adjacent tissue samples confirmed the high expression of MKI67, TYMS and LYPD3 at the protein level in LUAD (Figure 10C). We also evaluated the expression of these three genes in LUAD cell lines (including A549 and H1299) and the human bronchial epithelial cell line 16HBE using qRT‐PCR. The results showed upregulated expression of MKI67, TYMS, and LYPD3 in LUAD cell lines (Figure 10D). Given its strong association with the PCDS (AUC = 0.870) and its validated high expression in LUAD, MKI67 was selected for further in vitro experiments to study its role in LUAD development. MKI67 encodes the KI67 protein, which is an important proliferation marker in pathology, characterised by its absence in quiescent cells and heightened expression during periods of cellular proliferation, the KI67 labeling index has emerged as a benchmark for diagnosing cancer patients and evaluating their prognosis.

First, we successfully knocked down MKI67 in the A549 cell line (Figure 10E). Subsequently, CCK‐8 assays demonstrated that the proliferation capacity of A549 cells was significantly reduced in the MKI67 knockdown group compared with the control group (Figure 10F), suggesting that MKI67 may promote LUAD cell proliferation. Similarly, assays on colony formation revealed a notable reduction in colony quantity in the MKI67 knockdown group relative to the control group (Figure 10G). Additionally, transwell and scratch healing assays are revealed that reducing MKI67 levels markedly hindered the migration of A549 cells in contrast to the control group (Figure 10H,I). The findings validated MKI67's role in enhancing the growth and movement of A549 cells, suggesting its potential in promoting LUAD progression.

## 4 Discussion

Human health is greatly jeopardised by lung cancer, which ranks among the most prevalent malignant tumours globally [1]. In addition to traditional surgical treatment and chemotherapy, targeted therapies represented by EGFR inhibitors, ALK inhibitors, ROS1 inhibitors and immunotherapies represented by PD‐1/PD‐L1 inhibitors, and CTLA‐4 inhibitors are playing an increasingly critical role in the treatment of lung cancer [3, 4, 5]. Nevertheless, despite notable advancements in the realm of lung cancer treatment, the overall therapeutic outcomes and prognosis for most advanced LUAD patients continue to be unsatisfactory. In recent years, the rapid progress of bulk RNA‐seq and scRNA‐seq technologies have facilitated the identification of numerous tumour markers and therapeutic targets in lung cancer. As a result, an expanding repertoire of diagnostic and prognostic signatures has been constructed with the aim of enhancing the prognosis of individuals afflicted with this disease.

The regulatory mechanisms of PCD are highly complex, and mounting evidence indicating that PCD holds a pivotal position in various biological processes and has been associated with the progression and dissemination of malignant tumours [52]. The purpose of this study is to devise a PCD‐associated signature (PCDS) for LUAD, leveraging both scRNA‐seq and bulk RNA‐seq technologies. This endeavour aims to accurately predict the prognosis and treatment responsiveness of LUAD patients. Additionally, ML algorithms, which are widely used in the field of artificial intelligence based on the principles of statistics and computer science, have been extensively applied to tasks such as prediction, classification and clustering by extracting patterns, trends and correlations from large data sets through training and learning processes. Therefore, in this study, a robust prognostic signature, PCDS, was developed by leveraging 101 ML algorithms and key genes associated with PCD. In our study, we observed residual heterogeneity among multiple datasets even after accounting for batch effects. Validating models using independent data sets enhances reliability and demonstrates the generalisation ability of the model. Therefore, we utilised the TCGA‐LUAD data set as the training set and the GSE30219, GSE31210, GSE42127 and GSE68465 data sets as validation sets. To further assess the predictive performance of PCDS, we compared it with 140 previously published signatures. Encouragingly, in all four datasets, the C‐index values of PCDS were significantly superior to those of other signatures.

PCDS comprises 13 genes associated with PCD, namely MKI67, PTX3, TYMS, S100P, LAD1, LYPD3, SCNN1B, IL1A, SPIB, SLC34A2, CHPF, IER3 and CD79A. MKI67 is the encoding gene for KI67 protein, an important proliferation marker in pathology [53]. Its notable feature is the absence in quiescent cells and expression during cell growth [54]. The KI67 labeling index has developed into a benchmark for diagnosing cancer patients and evaluating prognosis [55]. PTX3 acts as a dissolvable inflammatory agent within the TME and is considered a possible indicator for inflammation [56]. This is also regarded as an inborn immune modulator linked to immune avoidance [57]. PTX3 has the ability to trigger the complement system, counteract pathogens, affect apoptotic cells and control inflammation [58]. TYMS, an essential folate‐dependent enzyme, is the sole producer of intracellular dTMP, vital for both DNA synthesis and repair processes [59]. Moreover, heightened levels of TYMS mRNA and protein have been correlated with adverse prognosis outcomes in various haematological malignancies and solid tumours [60]. The S100P gene is found in a range of cancers such as pancreatic, breast, prostate and lung cancers and is linked to negative clinical results [61, 62]. The LAD1 gene is responsible for producing collagen‐anchored filamentous protein in the basal layer [63]. This entity engages with actin cross‐linking proteins, facilitating the movement and growth of cancer cells via the EGF/ERK pathway, contributing to the formation of the cytoskeleton in breast cancer cells [64]. LYPD3, a cell surface protein known for its tumorigenic properties and high glycosylation, has been linked to carcinogenic impacts in numerous solid tumours [65]. An increase in LYPD3 levels is connected to the development of lung adenocarcinoma and unfavourable outlook [66]. IL1A encodes IL‐1α, which, along with IL‐1β, plays a dual role in malignant tumour progression [67]. Membrane‐associated IL‐1α enhances the immunogenicity of tumour cells, Monitoring the immune response in tumours and encouraging their shrinkage. However, the secreted form of IL‐1α has been shown to facilitate tumour invasion and angiogenesis [68]. SPIB, an Ets transcription factor unique to lymphocyte lineage, plays a role in the invasion of mesenchymal cells, connecting the spread of epithelial cancer to a lymphatic transcription program [69]. SLC34A2 functions as a phosphate transporter sensitive to pH levels, reliant on sodium. holds a pivotal position in lung cancer development. It has been reported that downregulation of SLC34A2 successfully suppresses lung cancer growth, reduces cancer cell proliferation and angiogenesis, and promotes apoptosis [70]. IER3 is a stress‐inducible gene that plays a pivotal role in cellular response to stress conditions [71]. Mounting evidence suggests that IER3 can exert either promoting or inhibitory effects on cancer development in various types of malignancies [72, 73].

By analysing the expression rates of these 13 genes, we calculated a risk score for every LUAD patient and classified them into groups of high and low risk. Examination of survival rates indicated a lower OS rate (OS) for the group at high risk, potentially associated with heightened activity of pro‐oncogenic pathways within this group. Notably, the high‐risk group exhibited a wider spectrum of gene mutations and showed elevated levels of chromosomal instability (CIN), resulting in significantly higher TMB scores. Conversely, the low‐risk group displayed a stronger immune response, enhanced antitumour immune capacity and a more favourable response to immunotherapy, culminating in improved prognostic outcomes. To enhance treatment response and prognosis for the high‐risk group, we identified potential efficacious small molecule drugs (e.g., SB‐743921 and ispinesib) specifically targeting this group using the CTRP2.0 and PRISM databases. Furthermore, the high‐risk group exhibited greater sensitivity to commonly administered chemotherapy and targeted therapy medications such as paclitaxel, gemcitabine, sorafenib and osimertinib.

In this study, the established PCDS demonstrated a significant correlation with prognosis and outperformed the previously established 140 models in terms of predictive efficacy, highlighting its broad potential as a complementary biomarker in clinical practice. However, there are still several unresolved issues. Due to budgetary and experimental constraints, we solely analysed public data sets without additional sequencing or collection of additional clinical data. The absence of our own independent data may result in a lack of external validation for our research conclusions. Relying on related data and lacking sufficient experimental validation could impact the universality and biological significance of our research findings. Further validation of the prognostic value of PCDS requires reliable prospective multicenter cohort studies. Moreover, we only experimentally validated three selected genes within PCDS, potentially leading to an insufficiently comprehensive exploration of the biological significance of PCD‐related genes. Additional wet lab experiments are needed to elucidate the precise biological functions and mechanisms of these 13 PCD‐related genes in LUAD cells, including in vitro and in vivo experiments. Third, evaluating and interpreting the immunological features of the model using bioinformatics methods pose a challenge. In this study, we employed mainstream, conventional bioinformatics approaches to reveal distinct tumour immune microenvironments in high‐ and low‐risk groups. However, the conclusions drawn in this study are correlational, making it difficult to elucidate the interactions between PCD and immune cells from a biological mechanism perspective, as well as to thoroughly describe the role of PCD in immune response reactions. Validation through experiments related to immunity in subsequent studies will help avoid oversimplifying the complexity of biological issues. Lastly, the validation of PCDS in predicting immune therapy response would benefit from the inclusion of more LUAD immunotherapy data sets.

In summary, by integrating scRNA‐seq and bulk RNA‐seq data and utilising a fusion of 101 machine learning algorithms, we successfully developed a highly promising PCDS in LUAD. This signature demonstrates the ability to predict patient prognosis and treatment response. The emergence of PCDS further elucidates the involvement of PCD in LUAD and provides potential opportunities for improving the prognostic outcomes of LUAD patients.

## Author Contributions

**Zipei Song:** conceptualization (lead), data curation (lead), formal analysis (lead), funding acquisition (lead), investigation (lead), methodology (lead), project administration (lead), resources (lead), software (lead), supervision (lead), validation (lead), visualization (lead), writing – original draft (lead), writing – review and editing (lead). **Weiran Zhang:** investigation (supporting), validation (supporting), visualization (supporting), writing – original draft (supporting), writing – review and editing (supporting). **Miaolin Zhu:** project administration (supporting), validation (supporting), visualization (supporting), writing – original draft (supporting), writing – review and editing (supporting). **Yuheng Wang:** software (supporting), validation (supporting), writing – original draft (supporting), writing – review and editing (supporting). **Dingye Zhou:** project administration (supporting), writing – review and editing (supporting). **Xincen Cao:** writing – original draft (supporting), writing – review and editing (supporting). **Xin Geng:** validation (supporting), writing – original draft (supporting). **Shengzhe Zhou:** writing – review and editing (supporting). **Zhihua Li:** data curation (lead), investigation (lead), project administration (lead). **Ke Wei:** data curation (lead), investigation (lead), project administration (lead), writing – original draft (lead), writing – review and editing (lead). **Liang Chen:** project administration (lead), validation (lead), visualization (lead), writing – original draft (lead), writing – review and editing (lead).

## Consent

Prior to their participation, all subjects provided their informed consent for inclusion.

## Conflicts of Interest

The authors declare no conflicts of interest.

## Supporting information


**Figure S1.** Preliminary processing of scRNA‐seq data. (A) The distribution of gene expression levels, sequencing depth, percentage of mitochondrial genes and percentage of red blood cell genes in the 12 samples. (B) The t‐SNE plot displayed the cell distribution of the 12 LUAD samples. (C) After dimensionality reduction and clustering, the t‐SNE plot showed that all cells were grouped into 16 clusters. (D) The bar plot depicted the distribution differences of cell types among the 12 LUAD samples. (E) The t‐SNE plot displayed the PCD activity of each cell.

**Figure S2.** Comparison of high and low PCD‐AUC groups in scRNA‐seq data. (A) Statistical analysis was performed on the cell communication quantity and cell communication intensity between the high and low PCD‐AUC groups. (B) The distribution of incoming and outgoing signal interaction strength in different cell populations between the high and low PCD‐AUC groups was examined. (C, D) Heatmaps depicting the strength of outgoing and incoming signalling pathways in different cell subgroups. (E) Differences in cell communication in the CCL pathway between the high and low PCD‐AUC groups. (F) Differences in cell communication in the MK pathway between the high and low PCD‐AUC groups.

**Figure S3.** Selection of key genes. (A, B) The PCA plots depict the sample distribution of LUAD bulk RNA‐seq data from the TCGA and GTEx databases before (A) and after (B) batch correction. (C) The heatmap displays the expression of 145 PCD‐related DEGs, identified through differential analysis, in normal and tumour tissues (*p* < 0.05, |log2FC| > 1.5).

**Figure S4.** Risk curves, survival scatter plots and expression heatmaps of 13 modelling genes were generated for the high‐ and low‐risk groups in TCGA‐LUAD (A), GSE30219 (B), GSE31210 (C), GSE42127 (D) and GSE68465 (E).

**Figure S5.** PCA plot and ROC curves incorporating clinical features. (A) The PCA plots show the distribution of patients from the high‐risk and low‐risk groups in the GSE30219, GSE31210, GSE42127 and GSE68465 datasets. (B) The ROC curves combining clinical features of the GSE30219, GSE31210, GSE42127 and GSE68465 datasets.

**Figure S6.** Immune cell infiltration landscape. (A) The scatter plot displays the correlation between risk score and immune score, stromal score, ESTIMATE score and tumour purity. (B, C) Seven algorithms were used to assess the association between PCDS score and immune cell infiltration.

**Figure S7.** Molecular docking pattern depicts the interaction conformations between proteins and small molecule drugs. (A) The molecular docking model illustrates the interaction between the MKI67 protein and the small molecule drug ispinesib. (B) The molecular docking model demonstrates the interaction between the TYMS protein and the small molecule drug SB‐743921.

**Figure S8.** IC50 values of common chemotherapy and targeted drugs were compared between the high‐ and low‐risk groups using the GDSC database.

**Figure S9.** Immunohistochemistry (IHC) images of the 12 modelling genes (MKI67, TYMS, LYPD3, PTX3, S100P, LAD1, SCNN1B, SPIB, SLC34A2, CHPF, IER3 and CD79A) obtained from the Human Protein Atlas (HPA) database (The HPA database does not provide IHC images for IL1A).

**Table S1.** Information of 2088 genes related to PCD.

**Table S2.** Differentially expressed genes between the high and low PCD‐AUC groups (|log2FC| > 0.7, *p* < 0.05).

**Table S3.** Total of 374 genes were obtained by integrating 150 genes strongly correlated with PCD and the differentially expressed genes.

**Table S4.** Total of 145 differentially expressed genes related to PCD were identified between LUAD and normal tissues using the GTEx and TCGA databases.

**Table S5.** The 25 key PCD genes used for model construction.

## Data Availability

The data sets analysed in this study are available in the TCGA repository (https://portal.gdc.cancer.gov/), and GEO (https://www.ncbi.nlm.nih.gov/geo/). Further enquiries can be directed to the corresponding authors.
