# Supplementary figures and images for: Unveiling Varied Cell Death Patterns in Lung Adenocarcinoma Prognosis and Immunotherapy Based on Single‐Cell Analysis and Machine Learning
Source: J Cell Mol Med. 2024 Nov 27;28(22):e70218. doi: 10.1111/jcmm.70218 (PMC11601877; doi:10.1111/jcmm.70218)

A

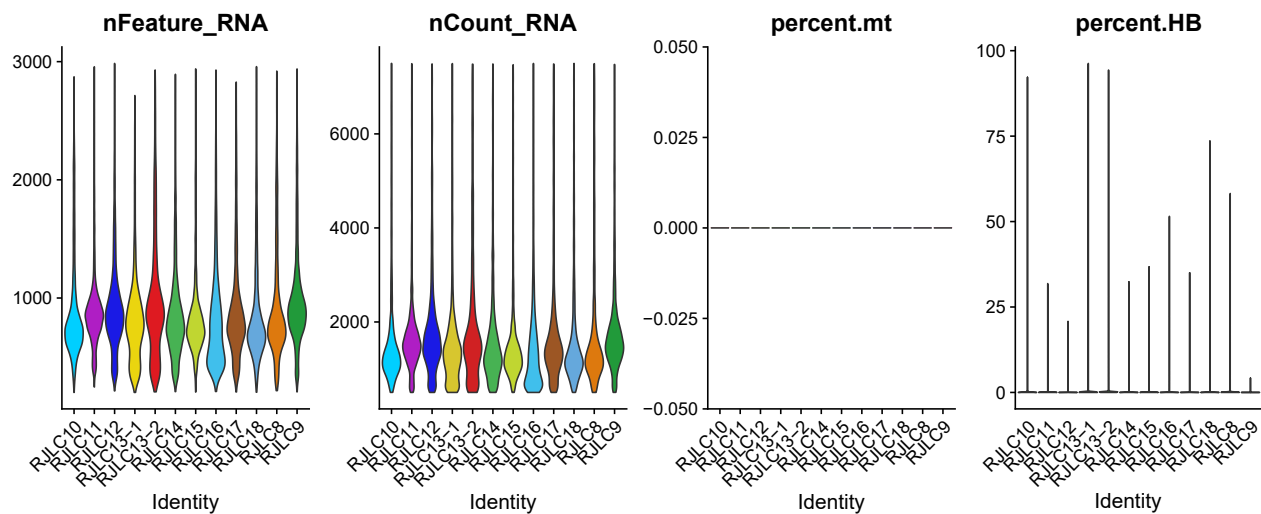

B

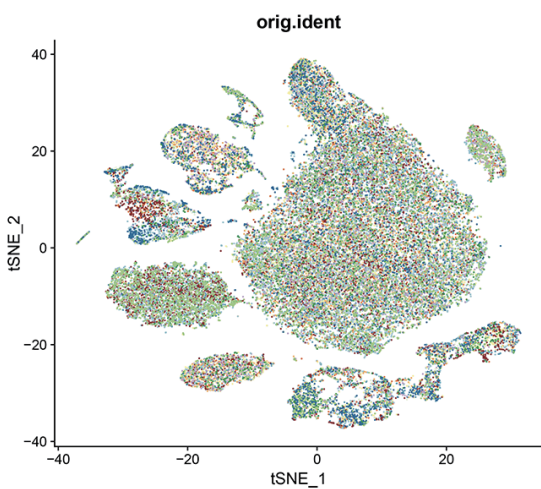

C

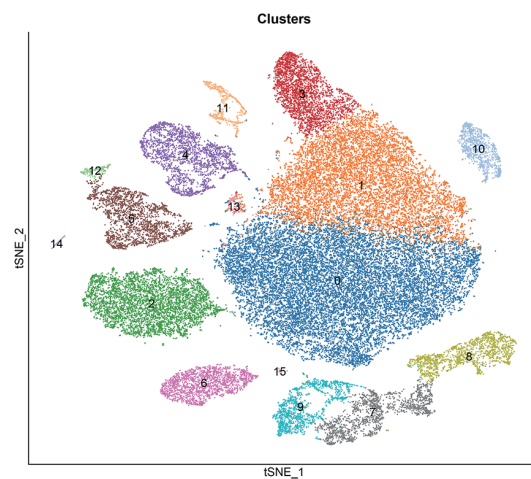

D

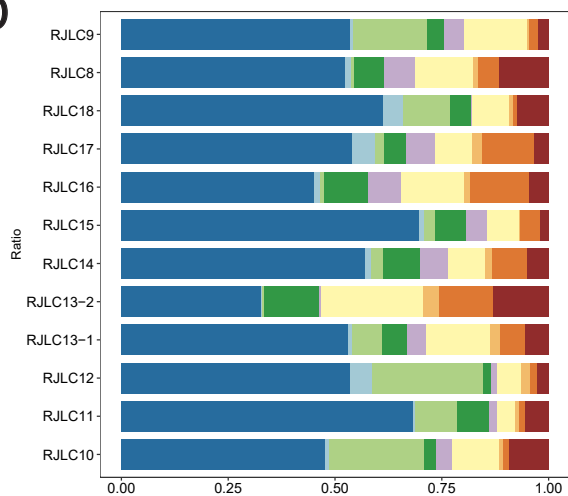

E

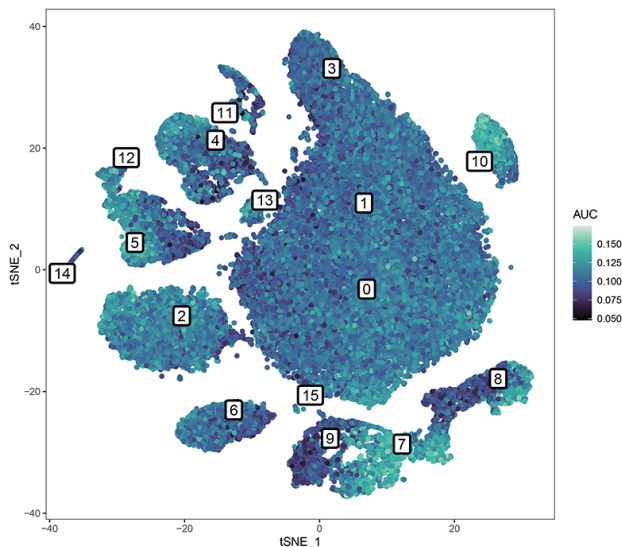

Supplement: Supplementary file 1 — Figure S1. Preliminary processing of scRNA‐seq data. (A) The distribution of gene expression levels, sequencing depth, percentage of mitochondrial genes and percentage of red blood cell genes in the 12 samples. (B) The t‐SNE plot displayed the cell distribution of the 12 LUAD samples. (C) After dimensionality reduction and clustering, the t‐SNE plot showed that all cells were grouped into 16 clusters. (D) The bar plot depicted the distribution differences of cell types among the 12 LUAD samples. (E) The t‐SNE plot displayed the PCD activity of each cell. [file JCMM-28-e70218-s009.pdf]

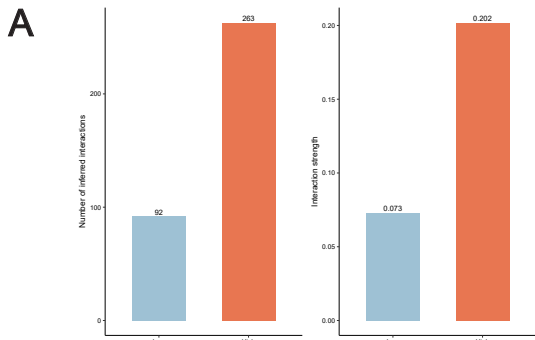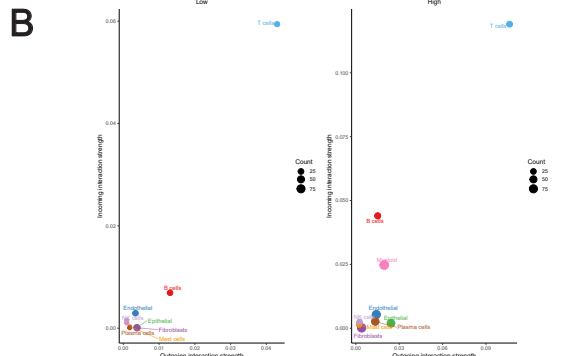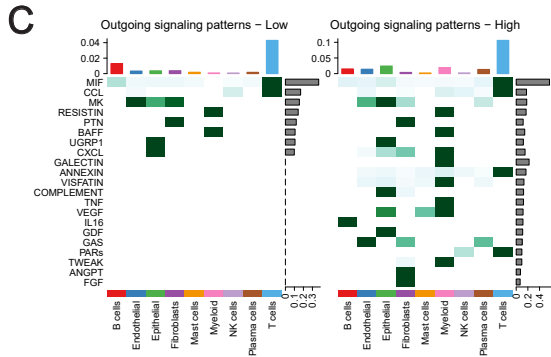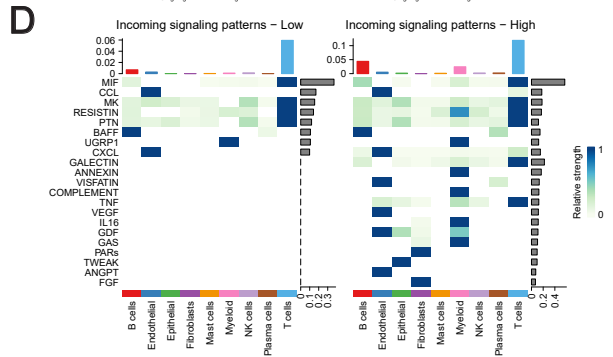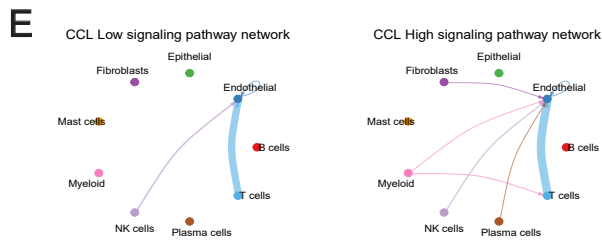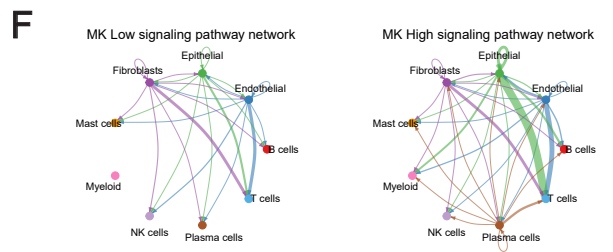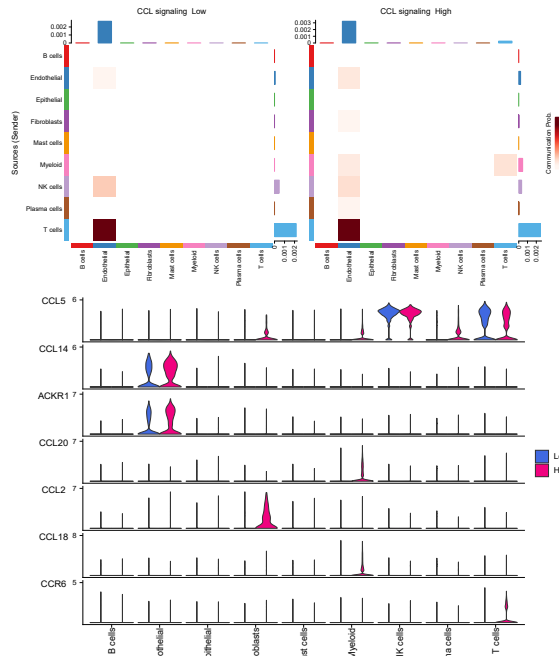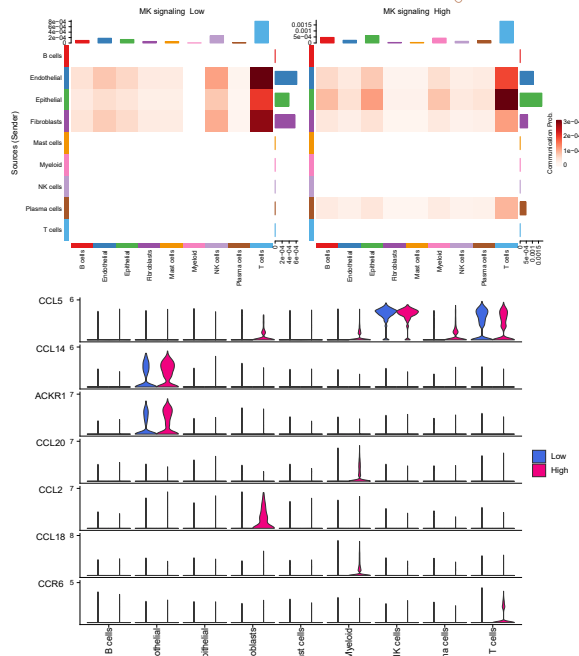

Supplement: Supplementary file 2 — Figure S2. Comparison of high and low PCD‐AUC groups in scRNA‐seq data. (A) Statistical analysis was performed on the cell communication quantity and cell communication intensity between the high and low PCD‐AUC groups. (B) The distribution of incoming and outgoing signal interaction strength in different cell populations between the high and low PCD‐AUC groups was examined. (C, D) Heatmaps depicting the strength of outgoing and incoming signalling pathways in different cell subgroups. (E) Differences in cell communication in the CCL pathway between the high and low PCD‐AUC groups. (F) Differences in cell communication in the MK pathway between the high and low PCD‐AUC groups. [file JCMM-28-e70218-s012.pdf]

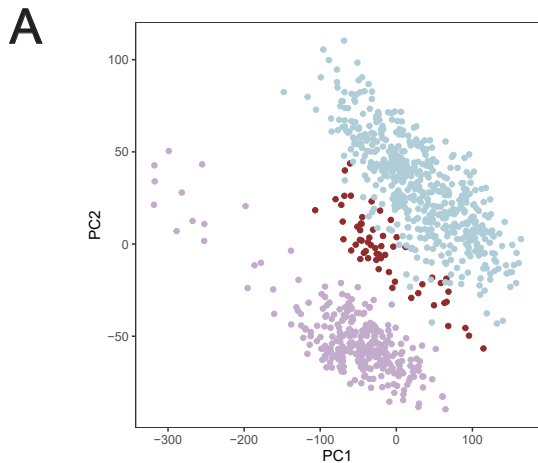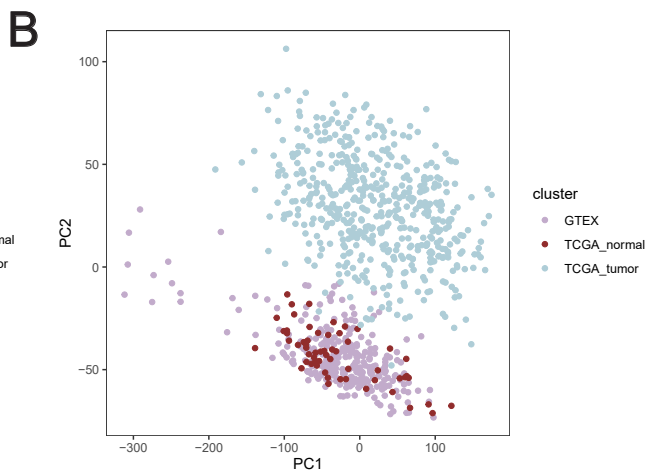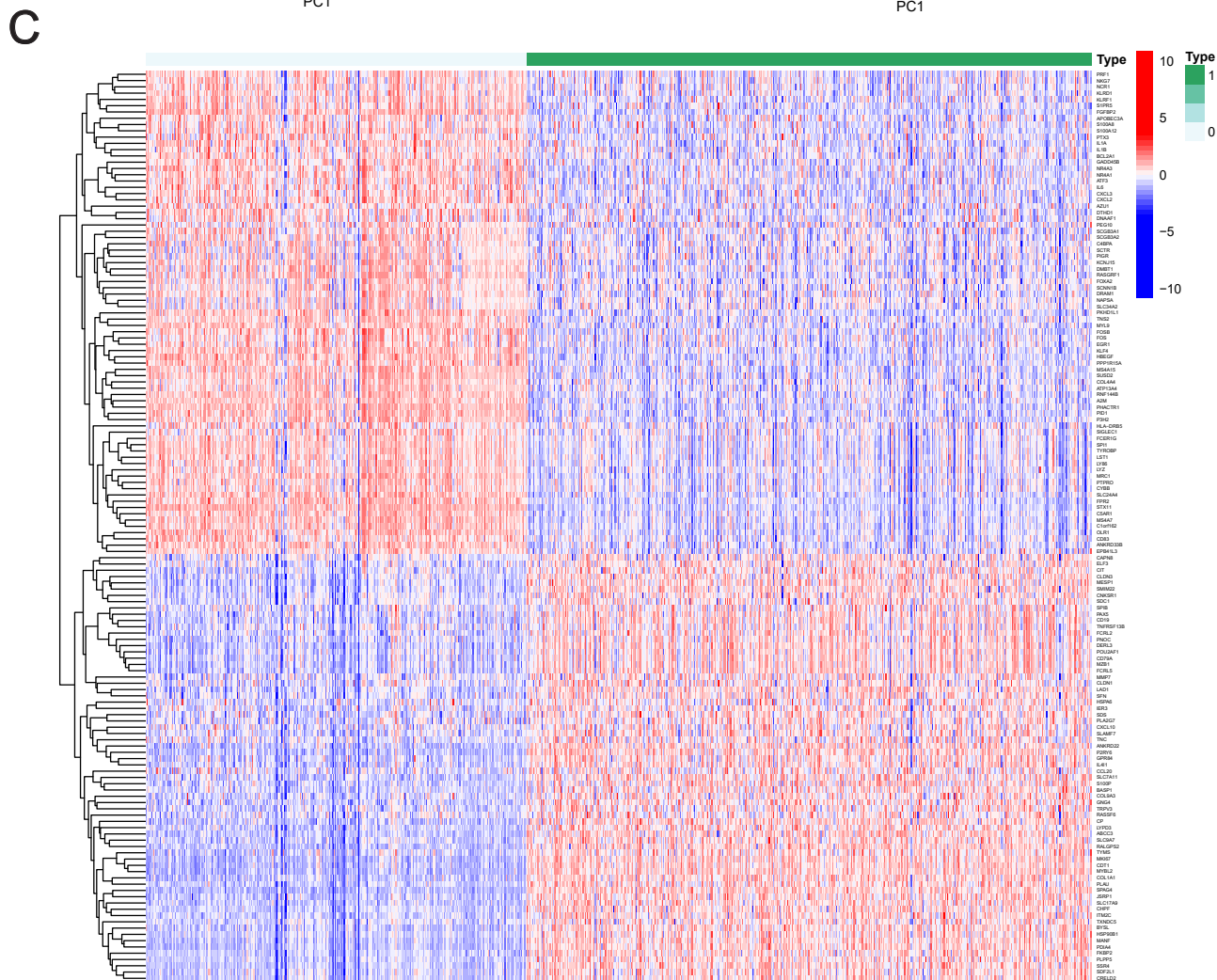

Supplement: Supplementary file 3 — Figure S3. Selection of key genes. (A, B) The PCA plots depict the sample distribution of LUAD bulk RNA‐seq data from the TCGA and GTEx databases before (A) and after (B) batch correction. (C) The heatmap displays the expression of 145 PCD‐related DEGs, identified through differential analysis, in normal and tumour tissues (p < 0.05, |log2FC| > 1.5). [file JCMM-28-e70218-s003.pdf]

A

## TCGA-LUAD

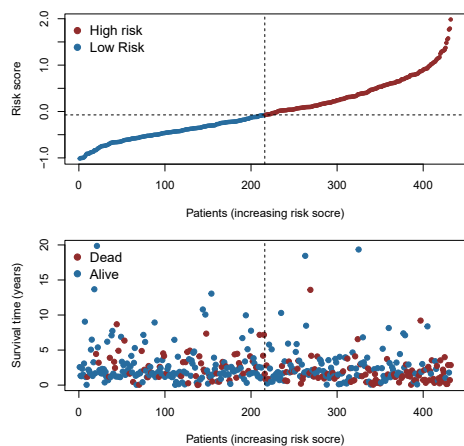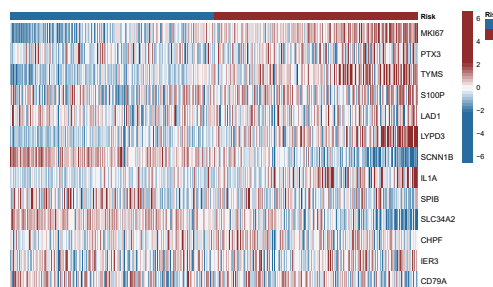

B

## GSE30219

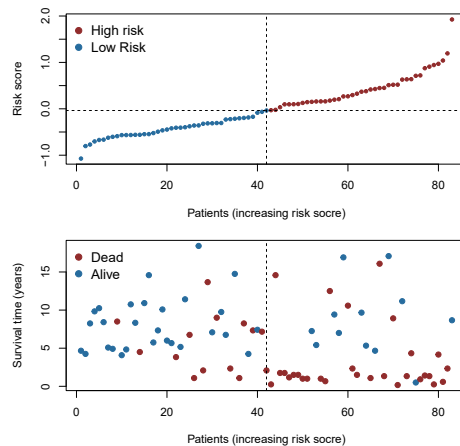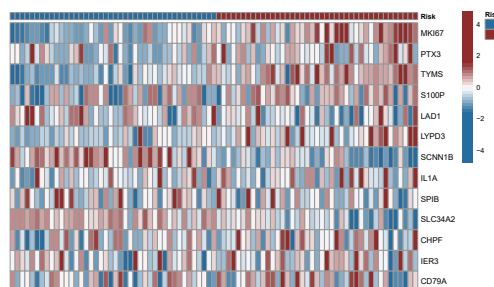

C

## GSE31210

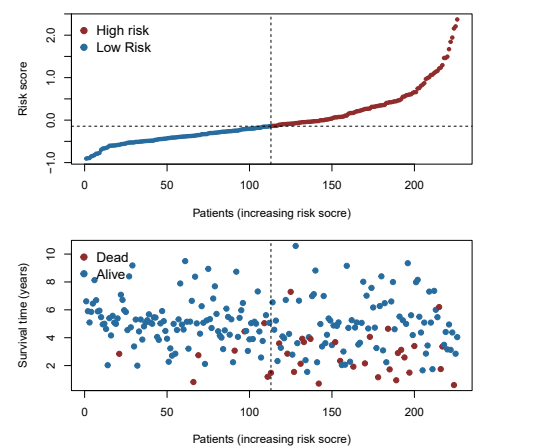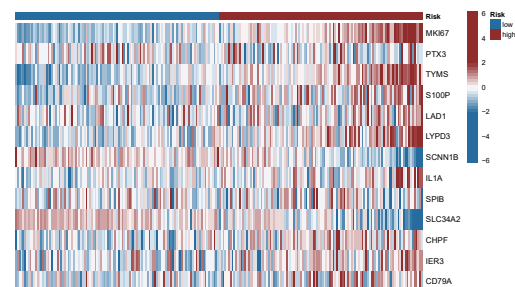

D

## GSE42127

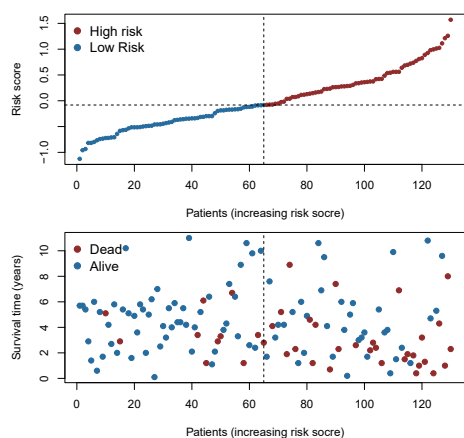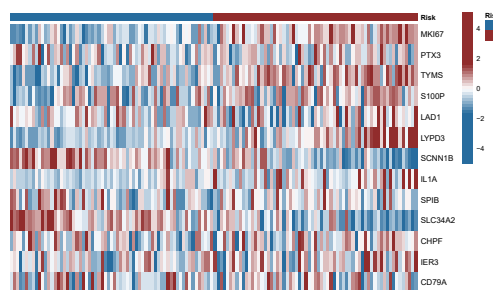

E

## GSE68465

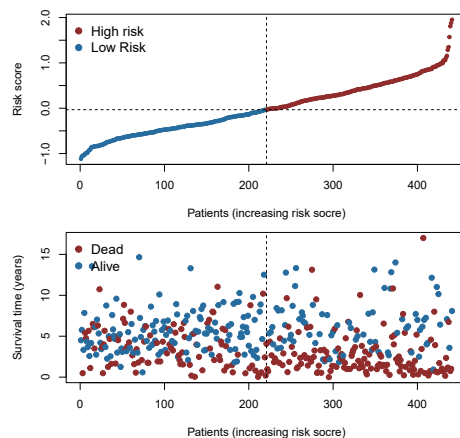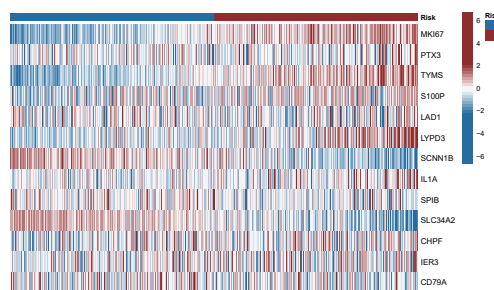

Supplement: Supplementary file 4 — Figure S4. Risk curves, survival scatter plots and expression heatmaps of 13 modelling genes were generated for the high‐ and low‐risk groups in TCGA‐LUAD (A), GSE30219 (B), GSE31210 (C), GSE42127 (D) and GSE68465 (E). [file JCMM-28-e70218-s014.pdf]

A

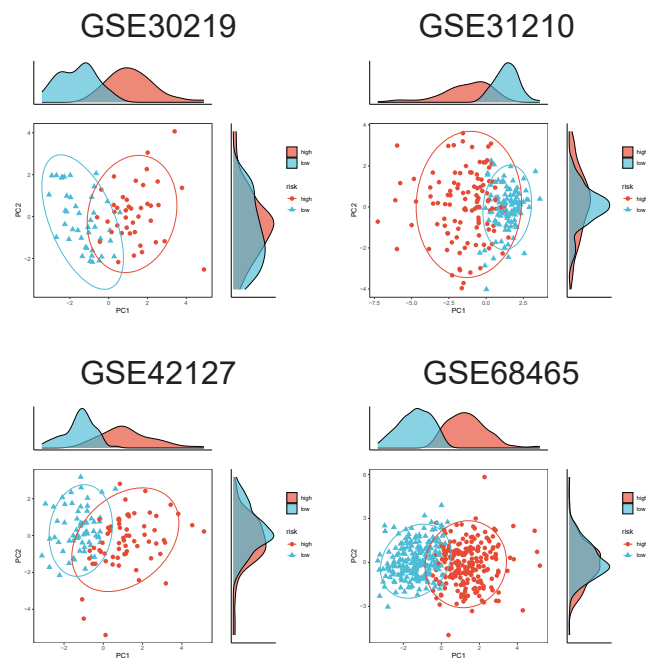

B

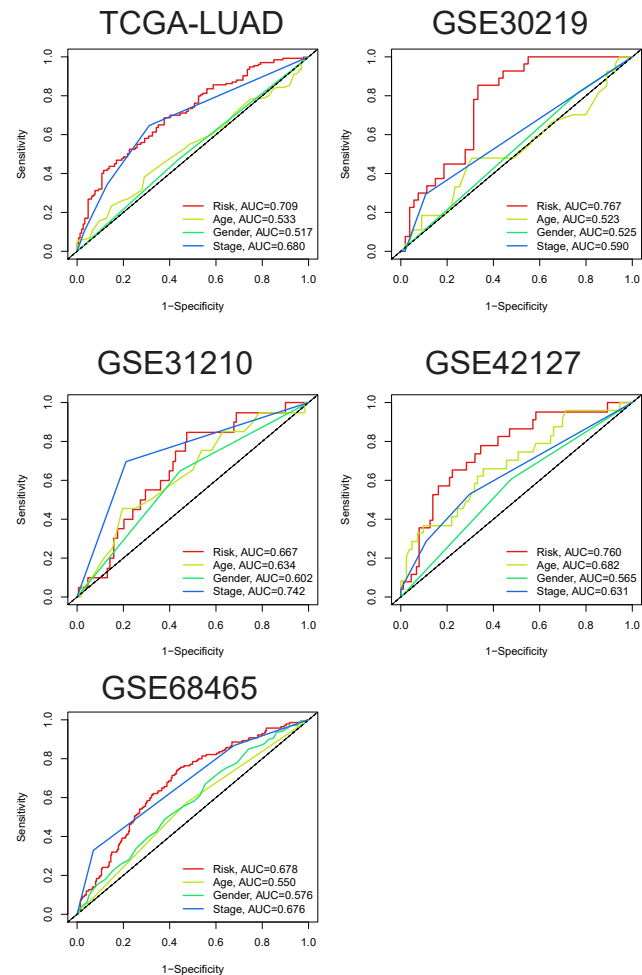

Supplement: Supplementary file 5 — Figure S5. PCA plot and ROC curves incorporating clinical features. (A) The PCA plots show the distribution of patients from the high‐risk and low‐risk groups in the GSE30219, GSE31210, GSE42127 and GSE68465 datasets. (B) The ROC curves combining clinical features of the GSE30219, GSE31210, GSE42127 and GSE68465 datasets. [file JCMM-28-e70218-s008.pdf]

A

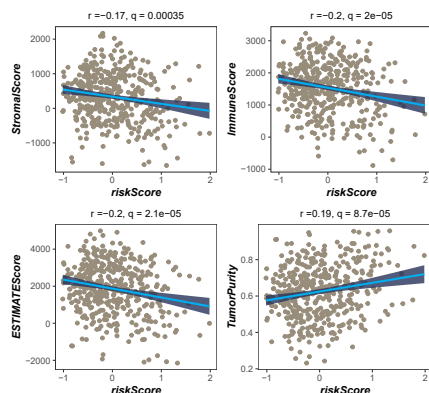

B

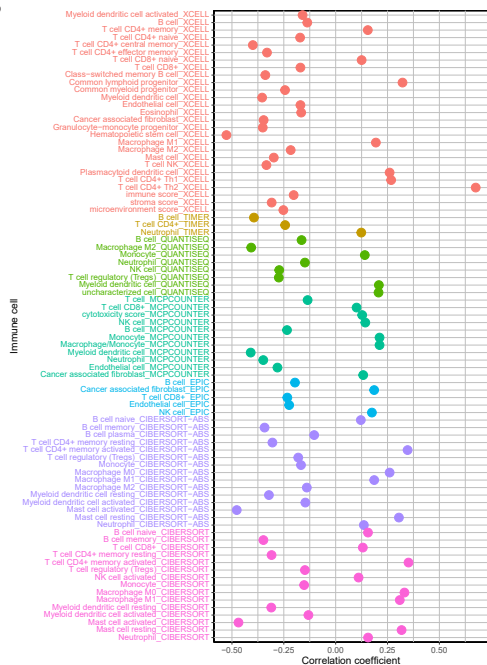

C

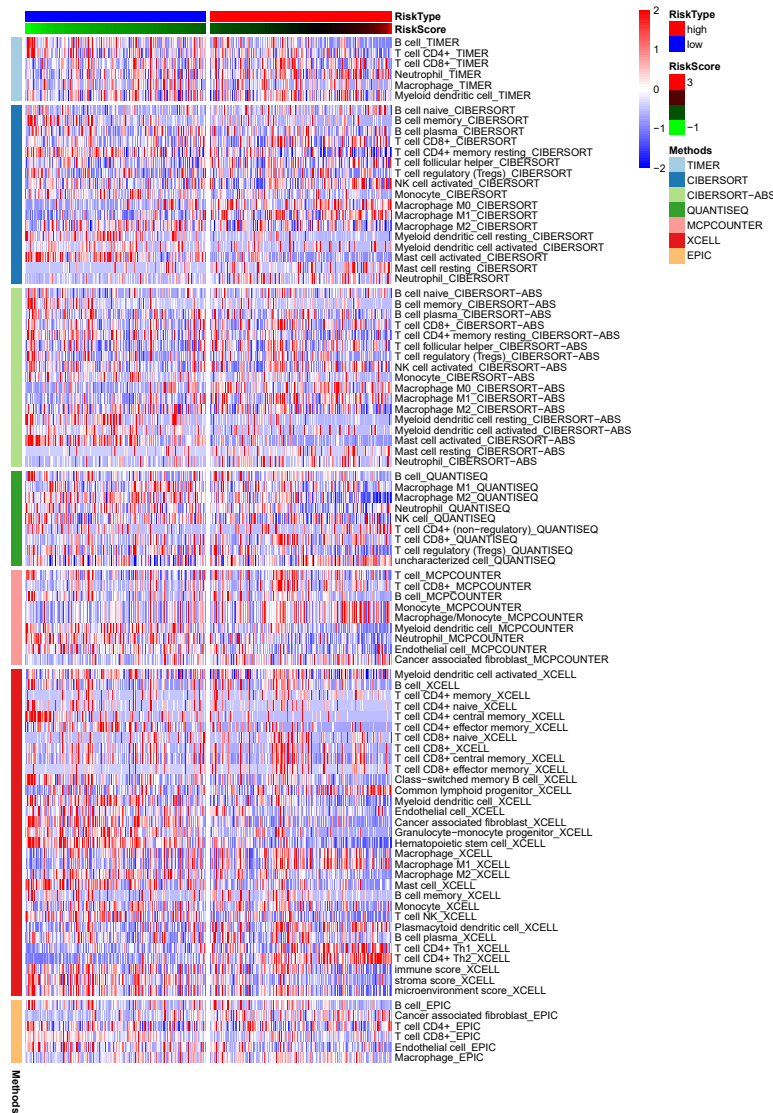

Supplement: Supplementary file 6 — Figure S6. Immune cell infiltration landscape. (A) The scatter plot displays the correlation between risk score and immune score, stromal score, ESTIMATE score and tumour purity. (B, C) Seven algorithms were used to assess the association between PCDS score and immune cell infiltration. [file JCMM-28-e70218-s007.pdf]

A

## Ispinesib-MK167

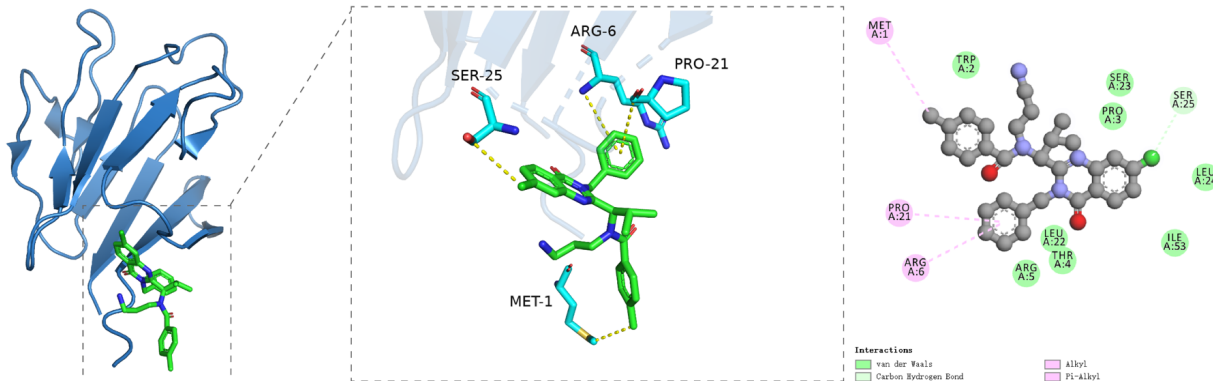

B

## SB-743921-TYMS

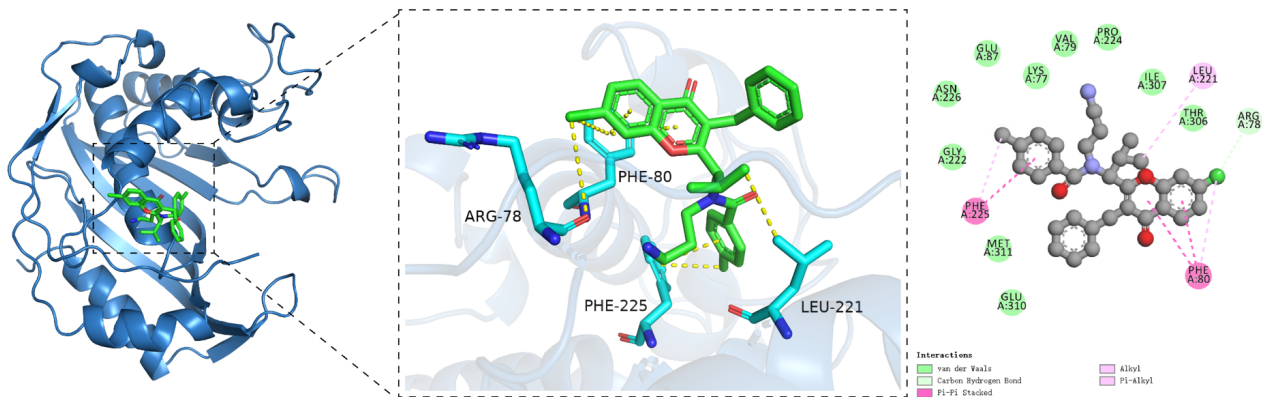

Supplement: Supplementary file 7 — Figure S7. Molecular docking pattern depicts the interaction conformations between proteins and small molecule drugs. (A) The molecular docking model illustrates the interaction between the MKI67 protein and the small molecule drug ispinesib. (B) The molecular docking model demonstrates the interaction between the TYMS protein and the small molecule drug SB‐743921. [file JCMM-28-e70218-s001.pdf]

**A**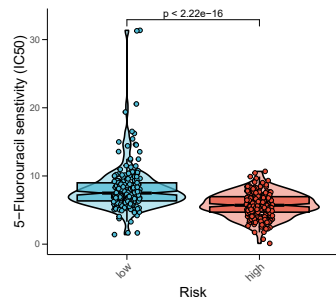**B**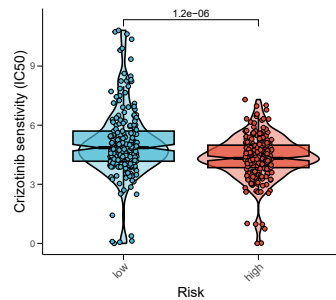**C**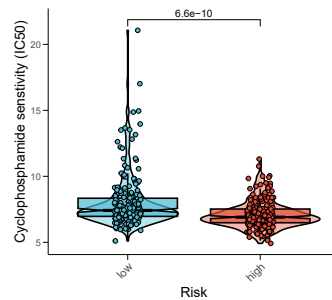**D**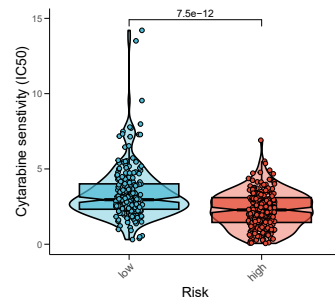**E**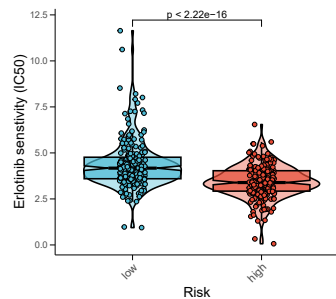**F**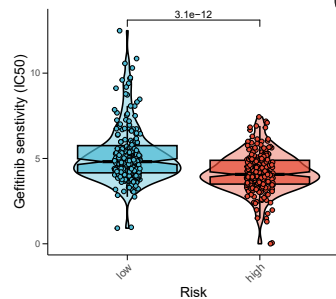**G**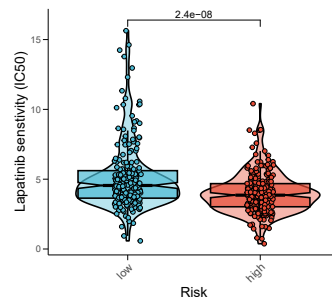**H**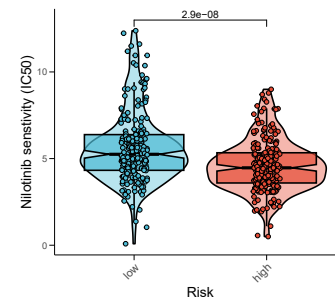**I**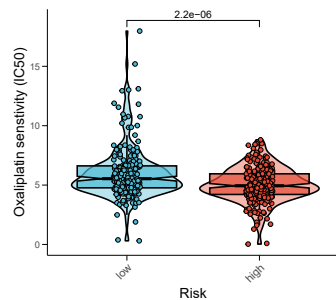**J**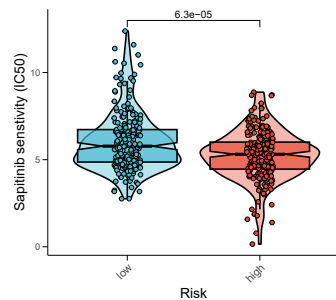**K**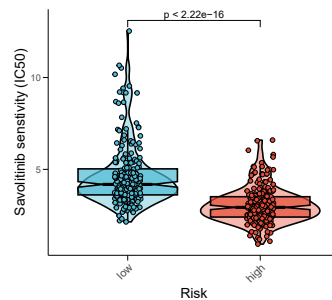**L**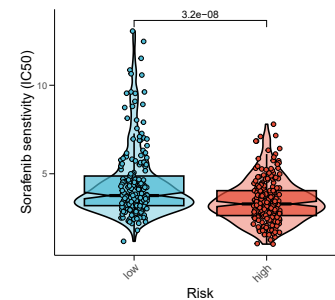

Supplement: Supplementary file 8 — Figure S8. IC50 values of common chemotherapy and targeted drugs were compared between the high‐ and low‐risk groups using the GDSC database. [file JCMM-28-e70218-s005.pdf]

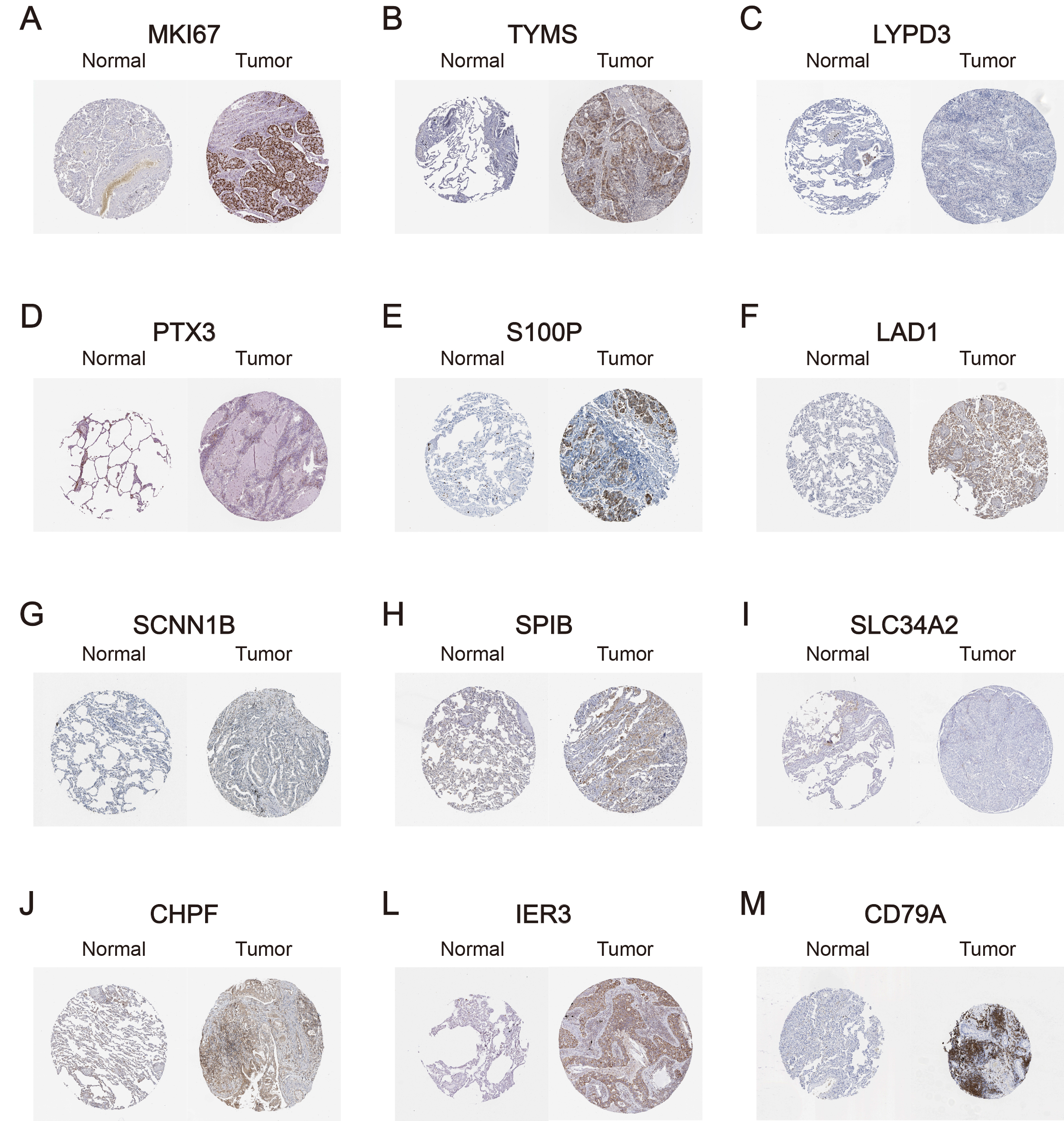

Supplement: Supplementary file 9 — Figure S9. Immunohistochemistry (IHC) images of the 12 modelling genes (MKI67, TYMS, LYPD3, PTX3, S100P, LAD1, SCNN1B, SPIB, SLC34A2, CHPF, IER3 and CD79A) obtained from the Human Protein Atlas (HPA) database (The HPA database does not provide IHC images for IL1A). [file JCMM-28-e70218-s013.jpg]
